# Nanoparticles as Anti-Microbial, Anti-Inflammatory, and Remineralizing Agents in Oral Care Cosmetics: A Review of the Current Situation

**DOI:** 10.3390/nano10010140

**Published:** 2020-01-13

**Authors:** Florence Carrouel, Stephane Viennot, Livia Ottolenghi, Cedric Gaillard, Denis Bourgeois

**Affiliations:** 1 Laboratory “Systemic Health Care”, University of Lyon, University Claude Bernard Lyon 1, EA4129, 69008 Lyon, France; stephane.viennot@univ-lyon1.fr (S.V.); denis.bourgeois69@orange.fr (D.B.); 2 Department of Oral and Maxillo-facial Sciences, Sapienza University of Rome, 00185 Rome, Italy; livia.ottolenghi@uniroma1.it; 3 Institut national de Recherche en Agriculture, Alimentation et Environnement (INRAE), Unité de Recherche 1268 Biopolymères Interactions Assemblages (BIA), 44316 Nantes, France; cedric.gaillard@inra.fr

**Keywords:** nanoparticles, oral cosmetics, toothpaste, mouthwash, biofilm, caries, periodontology, hypersensitivity

## Abstract

Many investigations have pointed out widespread use of medical nanosystems in various domains of dentistry such as prevention, prognosis, care, tissue regeneration, and restoration. The progress of oral medicine nanosystems for individual prophylaxis is significant for ensuring bacterial symbiosis and high-quality oral health. Nanomaterials in oral cosmetics are used in toothpaste and other mouthwash to improve oral healthcare performance. These processes cover nanoparticles and nanoparticle-based materials, especially domains of application related to biofilm management in cariology and periodontology. Likewise, nanoparticles have been integrated in diverse cosmetic produces for the care of enamel remineralization and dental hypersensitivity. This review summarizes the indications and applications of several widely employed nanoparticles in oral cosmetics, and describes the potential clinical implementation of nanoparticles as anti-microbial, anti-inflammatory, and remineralizing agents in the prevention of dental caries, hypersensitivity, and periodontitis.

## 1. Introduction

A nanomaterial is defined as “an insoluble or biopersistant and intentionally manufactured material with one or more external dimensions, or an internal structure, on the scale from 1 nm to 100 nm” [1]. To date, nanotechnologies and nanomaterials have been extensively employed and the potential for growth in nanomedicine appears significant. Three principal fields of application are particularly targeted: diagnosis, drug administration, and regenerative medicine [2]. The main domains of nanoparticles in the field of dentistry globally may involve the teeth-whitening, polishing pastes for the enamel surface, dental implant coatings, dental filling, antisensitivity agents, and the prevention of caries [3,4].

One sector is particularly relevant to consumers: cosmetics, much of which is based on nanoparticles (NP). Cosmetics is specified as whatever substance or preparation is planned to be in contact with external parts of the human body or with mucous membranes and teeth of the buccal cavity with a view, principally or exclusively, to changing their appearance, cleaning them, flavoring them, balancing body odors, keeping them or preserving them in a healthy condition [5].

Toothpaste is a cosmetic hygiene product requiring daily use. In the oral care market, toothpaste is the biggest part. The toothpaste market was estimated in 2018 to be worth USD 26.1 billion, and it is predicted to reach USD 37.0 billion by 2024 [6]. Various forms of toothpaste, such as pastes, powder, and gels, give more choice to consumers, thus propelling demand [7]. High product usage across all age groups and income is the prime element driving this segment. The use of cosmetic products containing nano-objects, such as dentifrices containing titanium dioxide NPs, is commonly used in periodontal health.

Mouthwash is expected to show profitable growth in the coming years because of the growing professional recognition of biofilm disorganization, and their increasing use by consumers linked to oral quality of life. The provision of flavored mouthwash associated with components labeled as natural should increase demand. The persistence of oral disorders such as periodontal disease, interdental bleeding, halitosis, and gingivitis, as well as the management of older people’s prostheses, should also contribute to the demand for medicated and non-medicated mouthwash.

It is rational to question the progress made by these NPs compared to existing technologies as well as the interest in the massive use of NPs in recent years in oral health cosmetic products. Some properties of nanoscale objects can confer specific behaviors, amplifying, in some cases, their chemical reactivity, their electronic or magnetic behavior, or their potential for penetration of living organisms. The knowledge and practices of nanotechnology in the field of oral hygiene products are progressing due to the emergence of new technologies that contribute to the improvement of the quality of care and broadening of the field of application of dentifrices and mouthwash.

In 2018, a catalog of all nanomaterials included in cosmetic products available on the market was produced by the European Commission. The catalog listed both nanomaterials used as preservatives, UV-filters, and colorants specifying reasonably predictable exposure conditions and the categories of cosmetics [8]. Of the 29 nanomaterials listed in this catalog, 3 were identified as colorants, 4 as UV-filters, and 22 as “other” functions, including oral hygiene products. These diverse nanomaterials have a major impact in individual prophylaxis, the prevention of caries, and periodontitis, as well as the quality of life of consumers.

A systematic literature search was conducted on three databases, namely PubMed, Web of Science, and Scopus. Articles in these databases were searched using the keywords (“nanoparticle” OR “nanomaterial” OR “nanotechnology”) AND (“nanoparticle” OR “nanomaterial” OR “nanotechnology”) AND (“toothpaste” OR “dentifrice” OR “oral care” OR “mouthwash” OR “mouthrinse”) AND (“metal” OR “metal oxide” OR “silver” OR “gold” OR “zinc” OR “titaniumdioxide” OR “copper oxide” OR “chlorhexidine” OR “ferumoxytol” OR “Scutellaria baicalensis” OR “chitosan” OR “hydroxyapatite” OR “Casein phosphopeptide–amorphous calcium phosphate” OR “bioactive glass”) AND (“anti-bacterial” OR “anti-microbial” AND “anti-inflammatory” OR “remineralization”). The research timeframe was from 2000 to December 2019. The selection procedure was performed by two reviewers, who evaluated the titles and abstracts of the articles identified in the electronic databases.

Our article provides a synthesis of the progress made by nanomaterials and their use in the oral hygiene market as anti-microbial and remineralizing agents in oral care cosmetics. It presents the main current knowledge of the biological and physico-chemical effects of nanomaterials. However, the health consequences of exposure to nanomaterials of cosmetic origin are not addressed. This inventory is not intended to be a complete catalog, but wants to point out that nanotechnologies in dental hygiene products touch a wide spectrum of applications, ranging from traditional toothpaste and mouthwash through remineralizing, bactericidal, or bacteriostatic agents.

## 2. Properties and Structural Features of Nanosystems (Nanomaterials and Nanoparticles)

Nanosized systems are of a great interest mainly due to their exceptional surface-to-volume ratio and following distinctive optical, physical, and chemical properties, and to their increased bioavailability towards cells and tissues [9,10]. The nanosystems classically used in oral care cosmetics can be globally classified regarding their composition and structure. Nanosystem composition can be inorganic (metals, oxides, or calcium/phosphate (Ca/P) salts), organic (chitosan NPs) [11,12], phosphatidylcholin/cholesterol liposomes [13], or inorganic–organic hybrids (silica NPs encapsulated chlorhexidine (CHX); colloidal hexametaphosphate salt of CHX)). The nanostructured materials, often called nanomaterials, are defined as solid systems with nanodomains embedded in a large, dense matrix. Such structure must be distinguished from “free” NPs often synthesized as stable dispersed colloids. Both are used in oral care cosmetics that include either dense materials such as toothpaste, or liquid dispersed systems, such as mouthwash, for which ingredient stability during processing and ageing is a key point. The vertebrate bone, which is well known for its exceptional biocompatibility properties, is a perfect example of a natural nanostructured system with hydroxyapatite organized as nanocrystals embedded in collagen fibrils [14]. Please note that all oral care applications have so far been described, to our knowledge, by using only pure NPs, as they need an intermediate dense or liquid system to keep them from aggregation that reduces the benefit of the nanosized effect. The relative recent capacity to synthesize purified “free” NPs allows the design of new performing systems with innovative physico-chemical and bifunctional properties, along with biocompatibility.

NP morphology and size distribution may also be key factors for innovative oral care applications, as they are described as strategical parameters in other application fields dealing with nanometer-size particles [15,16,17,18]. Indeed, the expected nanometer-size effect on NP properties is generally claimed to be more efficient, since the size distribution is narrow, and the shape is homogeneous. Size distribution and shapes are highly associated with synthetic routes and chemical compositions of the NPs. For a unique NPs composition, various morphologies can be produced: nanospheres, nanorods, necklaces, nanoprisms, nanostars, or nanowires for the most common ones. Transmission or scanning electron microscopy, as well as scanning probe microscopy, are the tools of choice to analyze NPs following synthesis. Also, dynamic light-scattering can be used to characterize the average particle diameter and polydispersity index of aqueous NP dispersions.

The stability of NPs, from synthesis to sterilization, storage processes, and use in biological environment, are also of interest regarding the availability to maintain nanosized distribution after synthesis and purification steps [19,20,21,22]. For example, consecutively to their synthesis and stabilization in a liquid environment, free drying or lyophilization, which is conventionally used to store NPs in a dried state, is well known to enhance more or fewer aggregation-irreversible mechanisms [23,24,25,26]. Then, the in situ characterization of NPs in the final product may be of great interest, as well as their characterization in the biological environment, where interaction takes place with the biological substances (proteins, membrane phospholipids, solid minerals (tooth, done), ions, pH, enzymes, etc.).

Therefore, the relevant properties in the achieved products (toothpaste and mouthwash) are dependent mainly on the nanosized dimension [3,4,12,27,28,29,30,31,32,33,34]. However, to our knowledge, no studies have focused on NPs ageing or reorganization in relation to their stability in the oral care field. An important aspect to keep in mind when surveying the clinical application of NPs is the ageing of NPs between synthesis and their distribution in oral care products after incorporation within other ingredients. The evolution of size distribution by ageing of NPs, and the consecutive variation of their properties, is dependent on synthesis and purification protocols, as well as conditions of storage and the process of incorporation in the liquids or pastes. A prospective exploration of this point may be relevant for a complete understanding of the real impact of nanometer size of additive NPs on the final oral care product.

## 3. Nanoparticles as Anti-Microbial and Anti-Inflammatory Agents in Oral Care Products

In 2017, oral disorders affected 3.47 billion people in the world and represented one of the three most common causes of global diseases [35]. Among the oral diseases, the most prevalent were dental caries and periodontitis. Together, they represent the most common infectious human disease in the world [36].

Even if caries and periodontitis are multifactorial diseases, the main etiological factor is the presence of pathogenic bacteria. These bacteria are organized within an extracellular matrix to form a bacterial biofilm [37]. In the biofilm, the bacteria are assembled to form a barrier that resists antibiotics and promotes chronic systemic infections [29,38]. Also, bacteria are 1000 times more resistant to anti-microbial treatment than planktonic organisms [39]. Moreover, in biofilms, bacteria can escape the immune system by producing superantigens [40].

To combat these bacterial infections, metal, metal oxide, and other NPs appear to be promising alternatives due to their distinct physio-chemical properties (Table 1). Even if some materials have a naturally anti-bacterial activity, their anti-bacterial activity is increased when their dimensions are reduced to the nanometer regime. This is attributed to the physical structure of NPs and to the increased surface-to-volume ratio that permits them to interact and penetrate bacteria [41].

### 3.1. Nanoparticles of Metals or Metal Oxides

**Figure 1:**
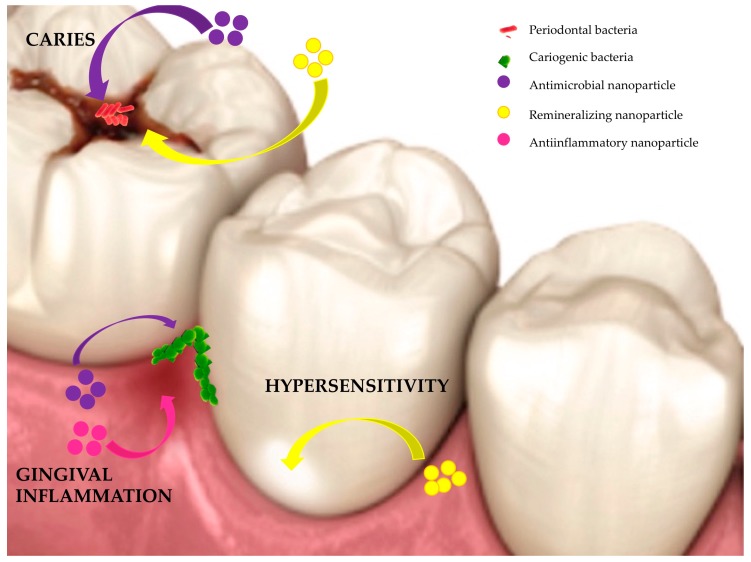
Action of nanoparticles (NPs) contained in oral care products.

Several metal or metal oxide NPs, such as gold (Au) NPs, silver (Ag) NPs, zinc oxide (ZnO) NPs, titanium dioxides (TiO_2_) NPs, copper oxide (CuO) NPs, and magnesium oxide (MgO) NPs, are known to have anti-microbial activities against both Gram-positive and Gram-negative bacteria and/or anti-inflammatory properties (Figure 1) [42].

#### 3.1.1. Anti-Microbial and Anti-Inflammatory Mechanism

To act as a anti-microbial agent, metal and metal oxide NPs must establish contact with bacteria via Van der Waals forces [43], electrostatic interactions [44], hydrophobic interactions [45], and receptor-ligands [46]. Then, the NPs pass through the bacterial membrane and organize themselves along the metabolic pathway that modifies the structure and function of the cell membrane. The interaction between NPs and bacterial components (enzymes, ribosomes, lysosomes, and DNA) will then cause oxidative stress and heterogeneous alterations, modifying the permeability of the cell membrane, disrupting the electrolyte balance, inhibiting certain enzymes, deactivating proteins and modifying gene expression [38,47,48]. Metal and metal oxide NPs use three mechanisms of action: metal ion release [49], oxidative stress [50], and non-oxidative mechanisms [51].

NPs act against bacteria mainly by producing reactive oxygen species (ROS) such as the hydroxyl radical (OH), the superoxide radical (O^−^), the hydrogen peroxide (H_2_O_2_), and the singlet oxygen (O_2_). In physiological conditions, the ROS are the product of oxygen metabolism and play a major role in cell signaling and cellular homeostasis [52]. However, if an inequality between the production and the elimination of ROS occurs, and if ROS are overproduced, then oxidative stress is observed [53]. The oxidative stress can provoke peroxidation, alter proteins, inhibit enzymes, and damage RNA/DNA [54,55]. The permeability of the bacterial membrane can be altered, which can deteriorate cell membranes [47,56,57]. Silver NPs are able to form OH, whereas [55] Au, ZnO, and MgO NPs generate H_2_O_2_, and TiO_2_ NPs form both OH and H_2_O_2_ [55].

Metal oxide NPs progressively release metal ions when they are in an aqueous medium. These metal ions can absorb cell membranes and then interact with functional groups (mercapto (-SH), carboxyl (-COOH), and amino (-NH) groups) of nucleic acids and proteins. These interactions have several consequences, including abnormal enzyme activities or modification of the structural cell, modification of the physiological processes, and inhibition of microorganisms [58]. Polivkova and colleagues demonstrated that silver nanowires prepared on polyethylene naphthalate released silver ions in solution and also have anti-bacterial effects [59]. However, other studies suggested that the main anti-microbial mechanism is not due to the release of metal ions by NPs, because metal ion solutions only provoke weak anti-bacterial activity [60,61]. In fact, silver NP cores have been found to preferentially interact in aqueous environments with various ligands to give oxidation, aggregation, or precipitation states to get a higher thermodynamic stability [62]. Hussein-Al-Ali and colleagues demonstrated that the interaction between superparamagnetic iron oxide and bacteria is mediated by penetration through the cell membrane, and through perturbation of the transfer of transmembrane electrons. Moreover, metal ions can be vectors for transporting anti-microbial substances [30].

Non-oxidative mechanisms can also explain the anti-microbial activity of NPs. Leung and colleagues proved the anti-microbial activity of three types of MgO NPs against *Escherichia coli* (*E. coli*) [51]. This anti-microbial activity was unrelated to oxidative stress. First, MgO NPs can damage cell membranes because pores are observed in cell membranes but neither MgO NPs nor magnesium ions were observed in microorganisms. Secondly, the intracellular ROS are low in quantity. Thirdly, MgO NP treatment does not induce lipid peroxidation, because the levels of lipopolysaccharide or phosphatidylethanolamine in the cell walls do not change significantly. Finally, intracellular protein levels associated with ROS are not affected, but several protein-associated metabolic pathways (amino acid, nucleotide, and carbohydrate metabolisms) are reduced. [51].

Several metal (silver, gold) and metal oxide (zinc oxide, titanium dioxide) NPs have anti-inflammatory properties [63]. To act as anti-inflammatory molecules, NPs must enter the cell through ion channels or pores. Depending on the size of the NPs, different cellular effects are observed [64]. When small NPs are at higher concentrations, most cell vesicles can endocytose them. Phagocytosis and macro-pinocytosis are achieved by macrophages and neutrophils [65]. In addition, if NPs are coated with plasma protein, then the corona protein interacts with the cell-surface receptors of macrophages or neutrophils [66]. The corona protein acts as a ligand for the M2 macrophage receptors. The anti-inflammatory macrophages of M2, essential in the assimilation of NP, are then activated. Finally, neutrophils organize extracellular traps (NETs) around NPs in response to exogenous stimuli (foreign particles or pathogens) and endogenous stimuli (cholesterol or uric acid). The interaction with pathogenic microorganisms and the response of inflammatory stimuli influences the formation of these NETs. The formation of NETs is dependent on RIPK-3 (protein kinase 3) enzymes that interact with receptors, as well as radicals of ROS [67].

#### 3.1.2. Silver Nanoparticles

Several synthetic or natural routes have been developed to produce silver NPs [68,69,70]. To avoid problems of toxicity during synthesis, biological methods are preferred. The use of bacteria, fungi, and plants, which do not produce toxic substances in their synthesis are used [71,72,73,74]. Plants have many advantages, as they need simple nutrients to grow and cover large surface areas, are economical, and are simple to process for Ag NPs synthesis over a range of sizes [75]. In recent years, Ag NPs have been synthesized from plants such as *Terminalia mantaly* extracts [70] or *Musa acuminata* colla flower [76]. The green synthesis of Ag NPs have also been obtained from vegetables such as *Ipomoea batatas* (sweet potato) [77] or from algae such as *Botryococcus braunii* [78].

Ag NPs are effective against Gram-positive and Gram-negative bacteria, and even against some antibiotic-resistant strains, but also against viruses and fungi (Table 1) [79,80,81,82]. In vitro, Ag NPs have an anti-microbial effect against Gram-negative bacteria such as *Acinetobacter* [83], *Escherichia* [84], *Pseudomonas* [85], and *Salmonella* [86]. These NPs also act against Gram-positive bacteria such as *Bacillus* [87], *Enterococcus* [88], *Listeria* [89], *Staphylococcus* [90], and *Streptococcus* [91]. The anti-bacterial activity of Ag NPs is influenced by the size of the NP. The biocompatility and the stability increase with decreasing size of Ag NP. The smaller Ag NPs have higher surface-area-to-volume ratio, which allows them to penetrate biological surfaces more readily [92,93,94]. These smaller Ag NPs interact with cell membranes and disorganize the lipid bilayer, causing the increase of the membrane permeability and bacterial lysis [95]. Ag NPs smaller than 30 nm demonstrated a strong anti-microbial activity against *Staphylococcus aureus* (*S. aureus*) and *Klebseilla pneumonia* (*K. pseumonia*), whereas Ag NPs with sizes ranging from 5 to 20 nm have a strong anti-microbial activity against *S. aureus* [96]. Therefore, small Ag NPs are more toxic than large particles, and even more when they are oxidized [97]. Indeed, the anti-bacterial activity of small Ag NPs (<10 nm) is mainly due to Ag^+^, whereas for large particles (>15 nm), the anti-microbial activity due to Ag^+^ is comparable to that of the particles, given that the release of Ag^+^ ions is proportional to the exposed nano silver surface area [98].

The morphology of Ag NPs is very important for anti-microbial activity. The colloidal morphology has higher anti-bacterial activity compared with polygonal, disk, prism, and hierarchical morphologies [99,100]. The concentration of silver NPs can also impact anti-bacterial activity [101]. Silver NPs smaller than 15 nm and with a concentration of 0.004% *w*/*w* have shown maximum effectiveness in preventing the growth of bacteria that cause unpleasant oral odors and tooth decay [87].

A synergic anti-bacterial effect against *Staphylococcus aureus* (*S. aureus*) and *E. coli* has been observed, in vitro, by combining Ag NPs with antibiotics such as amoxicillin, penicillin G, clindamycin, erythromycin, and vancomycin [40]. The conjugation of quinazolinone with Ag NPs demonstrated, in vitro, a higher anti-bacterial activity against *E. coli*, *Streptococcus pyogenes*, *Klebsiella pneumoniae* (*K. pneumoniae*), *Bacillus cereus* (*B. cereus*), and *Pseudomonas aeruginosa* (*P. aeruginosa*) as compared to quinazolinone alone [102]. Ag NPs can also inhibit, in vitro, the replication of viruses [81] such as HIV-1 [103], herpes simplex [104], and influenza [105].

Moreover, Ag NPs have anti-inflammatory properties [63]. For this, Ag NPs (i) decrease levels of vascular endothelial growth factor; (ii) reduce expression of the hypoxia-inducible factor 1α, which acts on bacterial destruction and regulates expression of pro-inflammatory genes; (iii) prevent the hypersecretion of mucins (mucus glycoproteins); and (iv) suppress the production of pro-inflammatory cytokines such as interleukin-12 and tumor necrosis factor α, and also provoke a decrease in the expression of the cyclooxygenase-2 gene at higher concentrations [63].

Due to their anti-microbial and anti-inflammatory effects, Ag NPs are used in healthcare products such as toothpaste, toothbrushes, and mouthwash [106]. The aim of such oral dental care is to fight against cariogenic and periodontal pathogens.

Ahmed and colleagues evaluated, in vitro, the action of toothpaste with and without Ag NPs against *Streptococcus mutans* (*S. mutans*) [31]. *S. mutans* is one of the main pathogens known to be involved in carious lesions [107]. Toothpaste containing Ag NPs had anti-microbial activity against *S. mutans* in vitro [31]. Using the agar-well diffusion method, the mean diameter of the zone of inhibition was measured as 20.14 ± 0.96 mm for this toothpaste, whereas no zone of inhibition was observed with the toothpaste without Ag NPs. In another in vitro study, Junevičius and colleagues demonstrated that Ag NP toothpaste had a lower effect against Gram-negative than against Gram-positive bacteria [108]. The minimal inhibitory concentration (MIC) affecting the growth of the fungus *Candida albicans* (*C. Albicans*) was 0.18 g/mL, whereas that affecting the growth of *S. aureus*, a Gram-positive bacterium, was 0.004 g/mL. For the Gram-negative bacteria, an MIC of 0.15 g/mL was observed for *K. pneumoniae* and *P. aeruginosa*, while no bactericidal effect was observed for *E. coli* or *Proteus mirabilis.* This toothpaste entirely inhibited the growth of *Enteroccosus faecalis* (*E. faecalis*) and *B. cereus* even when the toothpaste contained the lowest concentration of Ag NPs.

Mouthwash containing CHX is conventionally used for the treatment of plaque-induced gingivitis [109]. CHX is considered to be the gold standard for avoiding biofilm formation in addition to mechanical action due to toothbrushing [109]. However, the use of CHX can have various side effects [109,110]. For this, long-term therapy is not recommended [110]. Therefore, mouthwash containing Ag NPs could be an interesting alternative. The comparison of Ag NP mouthwash with CHX mouthwash, in a 6-month controlled clinical study, revealed no significant difference between these two products. However, a highly significant reduction in plaque index, gingival index, and papilla bleeding index after 2 and 4 weeks was observed [110]. One previous study had shown that CHX-containing mouthwash was statistically more effective than Ag NP mouthwash in vitro [111]. These differences could be explained by the differences in the composition of Ag NPs (a few silver ions contained in the hydrogen peroxide formulation), a different concentration of CHX (0.2%), and also differences in methodology (in vivo and in vitro study). Another use of mouthwash containing Ag NPs is to fight against colonization by certain bacteria resistant to drugs and also by fungi that cause infections in immunocompromised patients such as oral candidiasis (*Candida*-associated stomatitis) [112,113]. The in vitro anti-bacterial efficacity of an alcohol-free mouthwash containing a low concentration of colloidal Ag NPs (50–0.024 μg/mL) was demonstrated by Abadi and colleagues [114]. Using a challenge test on bacteria and fungi such as *S. aureus*, *S. mutans*, *P. aeruginosa*, *E. coli*, and *C. albicans*, they obtained measures of MIC ranging from 0.78 and 3.12 μg/mL, and measures of minimum bactericidal or fungicidal concentration (MBC or MFC) ranging between 1.56 and 12.5 μg/mL. At lower concentration of Ag NPs, the mouthwash killed all microorganisms tested. The addition of 30,000 μg/mL of ethanol to the mouthwash did not affect anti-bacterial activity.

Ag NPs are also used in impregnated toothbrush to reduce the number of the putative periodontal pathogens [115,116]. In a randomized controlled trial (RCT), do Nascimento and colleagues compared the use of toothbrushes with CHX-coated bristles, Ag-coated bristles, or conventional bristles over 30 days [115]. The toothbrush bristles with Ag NPs decreased the individual and total genome count in the supra- and subgingival biofilm. After 30 days of toothbrushing, the Ag-coated and CHX-coated bristles were able to reduce or maintain lower levels of bacterial counts of the putative periodontal pathogens such as *Porphyromonas gingivalis* (*P. gingivalis*), *Treponema denticola* (*T. denticola*), *Tanerella forsythia* (*T. forsythia*), *Prevotella intermedia* (*P. intermedia*), *Prevotella nigrescens* (*P. nigrescens*), *Fusobacterium nucleatum* (*F. nucleatum*), and *Parvimonas micra*. The total genome counts in supragingival or in subgingival biofilm was not decreased by CHX. However, in supragingival biofilm, CHX reduced the individual genome counts for most of the target pathogens [115].

Although Ag NPs appear to be very beneficial, one should also consider the adverse effects. Gaillet and Rouanet indicated that Ag NPs in toothpaste and other products could be responsible for inflammation of the gastrointestinal tract [117]. The main toxic effects observed were weight loss, disruption of blood biochemistry, and dysfunction of liver enzymes [117]. However, since toothpaste is not indicated for systemic use but for topical use, it is unlikely that adverse effects would be observed in such small quantities.

#### 3.1.3. Gold Nanoparticles

In recent years, research on NP synthesis has mainly focused on green synthesis using bacteria [73,118] and plants [119,120,121,122]. For example, the marine extremophilic bacteria *Pseudoalteromonas* was used to obtain Au NPs with non-cytotoxic, non-genotoxic and non-oxidative stress generated over a range of concentrations but provoking alterations in DNA methylation and in the expression of DNA methyltransferase genes [118]. Plants such as *Curcuma wenyujin* extract [119], *Chenopodium formosanum* shell extract [120], *Dillenia indica* leaf aqueous extract, or *Annona muricate* leaf extracts were also used [123]. Au NPs were also obtained using *Coffea arabica* [124].

Au has a weak anti-microbial effect against many microorganisms such as bacteria and fungi (Table 1) [123,125,126,127,128]. Conjugation with tetracycline [129] or with ampicillin [130] can enhance anti-bacterial activity. Moreover, Au NPs have anti-inflammatory action by (i) reducing ROS production; (ii) decreasing lipopolysaccharide-induced cytokine production such as interleukin (IL)-1β, IL-17, tumor necrosis factor (TNF)-α; and (iii) modulating mitogen-activated protein kinase and phosphatidyl inositol 3-kinase pathways [63].

The use of Au NPs in oral pathology remains uncommon [125], although they are easily prepared by the co-precipitation technique and are less toxic than metallic nanomaterials such as Ag NPs [131]. Essentially, as Au is inert, it represents the NP of choice to conjugate with various biomolecules and ligands to target microorganisms [132]. The effect is similar to the one observed with antibiotics such as mupirocin [133,134]. Hernández-Sierra and colleagues used NPs of Ag and Au of 25 nm, 80 nm, and 125 nm average sizes. The results indicate that in vitro, a higher concentration of Au NPs than that of Ag NPs is required to observe bacteriostatic and bactericidal effects on *S. mutans* [135]. Similar results to this study were observed by Junevičius and colleagues [108]. They compared, in vitro, toothpaste containing Ag NPs and Au NPs and observed that they were less active against Gram-negative than against Gram-positive bacteria. Moreover, Ag NP-containing toothpaste had a stronger effect on Gram-negative bacteria and a broader anti-microbial effect compared to toothpaste containing Au NPs. This lower anti-microbial effect for Au NPs compared with Ag NPs could explain the low use of Au NPs in oral care products. Au NPs are also used in drug delivery applications [136].

However, Au NPs are also incorporated between toothbrush bristles [125]. In addition to increasing mechanical elimination of plaque, gold has significantly reduced periodontal disease, because of its anti-bacterial action [137]. However, to our knowledge, no study concerning the action of the toothbrush containing the Au NPs bristles was conducted.

Regarding adverse effects of Au NPs, toxicology remains unclear. However, in one review, Jia and colleagues concluded that according to the range of concentrations, Au NPs are toxic when they are used in biological systems [138]. In vitro research demonstrated that Au NPs, having penetrated bacteria, will cause synthesis of ROS. This oxidative stress induces a new cytotoxicity that will damage DNA, cause cell death by apoptosis and necrosis, and cause cell-cycle arrest. As ROS production is known as a common mechanism of cytotoxic effects, therefore Au NPs can induce cytotoxic effects [138]. However, recently, Gunduz and colleagues studied the exposition of cultured vascular endothelial cells to non-lethal Au NPs [139]. They demonstrated that once the maximum number of intracellular NPs was reached, there was constant depletion but no cell death. This depletion reduced endoplasmic reticulum stress. This report suggests that in vitro, NPs uptake is actively regulated by cells and the impact of NPs exposure in the long-term [139]. In vivo, the inhalation of Au NPs by Sprague–Dawley rats during 6 h/day or 5 days/week for 90 days revealed that dose-related changes were observed only in the lungs [140]. Therefore, as it is well acknowledged that in vitro and in vivo results can diverge, other in vivo studies are necessary for more reliable conclusions.

#### 3.1.4. Zinc Oxide Nanoparticles

ZnO NPs biosynthesis was carried out using plant extracts such as *Ocimum tenuiflorum*, *Trifolium pretense*, or *Agathosma betulina* [122], *Menthaa pulegium* leaf extract [141], *Crocus Sativus* petal extract [142], or *Laurus nobilis* plant extract [143]. Another ZnO NP synthesis is the thermal decomposition of Zn-metal organic frameworks. Hajiashrafi and colleagues prepared ZnO NPs by thermal decomposition of prepared MOF-5 with and without tri-ethylamine using solution and solvothermal methods [144].

ZnO NPs fight against bacteria [142,145,146], viruses [147], and fungi [148]. The anti-microbial activity of ZnO NPs (Table 1) depends on synthesis techniques, physico-chemical characteristics, evaluation tools, and techniques used to generate three-dimensional structures [149,150]. In vitro, ZnO has anti-bacterial properties that increase when ZnO is in NP form due to the increasing of surface-area-to-volume ratio [151,152]. It was shown that ZnO NPs were able to damage *E. coli* bacteria by cellular internalization with a Gram-negative triple-membrane disorganization causing increase in membrane permeability [153]. Anti-bacterial effects on Gram-negative and Gram-positive bacteria have been demonstrated in vitro [142,145,146]. More recently, it was shown that polyethylenglycol-encapsulated ZnO interacts strongly with the citric acid and lactic acid, leading to a bacterial inhibition effect against *S. aureus* and *E. coli* [142,154], and *S. mutans* and *Lactobacillus* in dental plaque [155]. The anti-microbial effects of ZnO are similar to Ag NPs because the smaller the NPs are, the larger the surface area becomes. Therefore, the anti-microbial effect becomes stronger due to its capacity to react with more molecules per unit of surface. However, in vitro, compared with Ag NPs (0.1 mg/mL), a higher concentration of ZnO NPs (size 15–20 nm; surface area 47 m^2^/g) is necessary to observe growth inhibition (0.5–2.5 mg/mL) and killing effects (>2.5 mg/mL) against several pathogens [156]. ZnO NPs demonstrated, in vitro, an anti-bacterial activity against bacteria known to be implicated in gingivitis and periodontitis such as *Aggregibacter actinomycetemcomitans* (*A. actinomycetemcomitans*), *P. gingivalis*, *P. intermedia*, and *F. nucleatum* [157]. ZnO NPs are commonly used as anti-bacterial agents in oral care products such as toothpaste and mouthwash [158].

Moreover, another interesting property, to fight against gingivitis or caries, is the anti-inflammatory activity of ZnO NPs in response to pathogens [63]. ZnO NPs reduce inflammation by (i) blocking the production of pro-inflammatory cytokines such as IL-1β and IL-18 by inhibiting the necrosis factor kB and caspase 1 in activated mast cells and macrophages; (ii) inhibiting mast cell proliferation by increasing p53 and decreasing thymic stromal lymphopoietin production related to IL-13, a TH2 cytokine, along with IL-1 and tumor necrosis factor-α; and (iii) suppressing lipopolysaccharide-induced cyclooxygenase-2 and inducible nitric oxide synthase expression [63,159].

The incorporation of ZnO NPs in toothpaste and mouthwash enables the fighting of gingivitis due to bactericidal effects [32,160]. Toothpaste containing ZnO has also been demonstrated to have, on an extracted tooth, a positive effect on the dentin by reducing demineralization [161]. Moreover, due to its anti-bacterial and anti-gingivitis action, Zn has been incorporated into bioactive glass toothpaste. Increasing the quantity of Zn in toothpaste could result in a better anti-bacterial and anti-gingivitis effect [162]. By liquid dilution method, Hernandez-Sierra and colleagues revealed that the anti-microbial effect of ZnO NPs against *S. mutans* was lower than of Ag NPs [135]. Although it has been demonstrated that even if Ag and ZnO NPs have an anti-microbial effect against *S. mutans*, their mechanism is still not well known.

Mouthwash containing zinc salts (zinc gluconate, zinc chloride) or ZnO NPs presents high anti-bacterial activity against *S. mutans*, in vitro [163,164]. ZnO NPs also act against *Streptococcus sanguis* known to be implied in caries and periodontal diseases [165]. The combination of Ag Nps with ZnO NPs demonstrated a synergistic anti-bacterial effect [166]. Therefore, to control the formation of dental plaque, very low concentrations of Ag/ZnO NPs are necessary [135]. Mouthwash containing a low concentration of ZnO NPs have high anti-bacterial activity against *Streptococcus* in the mouth [167]. Similar results were obtained by Kachoei and colleagues [167]. They proved, in vitro, that mouthwash containing Ag/ZnO NPs was an effective anti-microbial agent. Compared to those containing ZnO NPs, chlorhexidine 0.2% and sodium fluoride 0.05%, mouthwash containing Ag/ZnO NPs demonstrated a higher anti-microbial activity against *S. mutans*. Therefore, to avoid plaque accumulation, mouthwash containing ZnO NPs could replace chlorhexidine 0.2% and sodium fluoride 0.05%. However, chlorhexidine 0.2% showed higher anti-microbial activity against *S. mutans* compared to different concentrations of ZnO NPs [167]. One problem associated with the use of ZnO NP mouthwash is enamel discoloration. Eslami and colleagues concluded that extracted teeth immersed in ZnO NPs had the most severe color change, followed by CuO NPs, Ag NPs, TiO_2_ NPs, and chlorhexidine, successively [168].

ZnO NPs represent a biologically safe material that does not exhibit toxicity to human cells [152]. Compared with their micron equivalents, ZnO NPs are more toxic for bacteria [169].

#### 3.1.5. Titanium Dioxide Nanoparticles

Recent research focused on the green synthesis of TiO_2_ NPs using plants such as *Azadirachta indica* leaf extract [170], *Glycyrrhiza glabra* [171], *Carica papaya leaves* [172], and *Cassia fistula* [173].

TiO_2_ is known to possess this anti-microbial activity in vitro (Table 1) [174,175]. It can fight against bacteria and viruses [176]. TiO_2_ NPs are of great interest due to their low cost, high stability, high photocatalytic activity, and reusability [48,177,178]. The bioactivity of TiO_2_ NPs is linked to the surface of contact and/or volume that is increased by decreasing the particle size, particularly in this case the thickness (<100 nm), which authorizes intensified interaction with molecules and proteins of the cellular membranes and a lesser total of substance [38,179,180,181]. Compared to Ag and Cu NPs, few studies on TiO_2_ anti-bacterial activity have been conducted.

The anti-microbial activity of TiO_2_ is due to its crystal structure, shape, and size [182]. To ensure the best anti-microbial activity, the anatase form of TiO_2_ NPs and the excitation by UV light are necessary. The photocatalysis of TiO_2_ NPs provokes: (i) the peroxidation of polyunsaturated phospholipid component of bacterial lipid membrane; (ii) loss of respiratory activity; and (iii) cell death [183]. Tsuang and colleagues demonstrated, in vitro, that TiO_2_-mediated photocatalytic had microbial activity against obligate anaerobes (*Bacteroides fragilis*), facultative anaerobes (*E. coli*, *S. aureus*), and obligate aerobes (*P. aeruginosas*) [184]. In the absence of UV light, TiO_2_ NPs of about 18 nm and a surface area of 87 m^2^/g demonstrated a bacteriostatic activity for concentration ranging from 1.0 to 2.5 mg/mL against *E. coli* and multi-resistant *S. aureus* and a bactericidal activity for concentration greater than 2.5 mg/mL. Against oral pathogens such as *A. actinomycetemcomitans*, *P. gingivalis*, *P. intermedia*, and *F. nucleatum*, a bacteriostatic activity is observed, in vitro, for concentrations ranging from 0.25 to 2.5 mg/mL, and bactericidal activity is demonstrated for concentration greater than 2.5 mg/mL [157].

De Dicastillo and colleagues developed TiO_2_ NPs from the combination of electrospinning and atomic layer deposition processes [174]. They obtained spherical, hollow, anti-microbial nanostructures with a shell of 17 nm thickness. These hollow, calcined TiO_2_ NPs presented better anti-microbial activity than commercial TiO_2_ NPs, against multidrug-resistant bacteria such as *Escherichia coli* and *S. aureus* [174]. The coupling of TiO_2_ NPs with a second metal strongly modified the photocatalytic and biocidal activity of the obtained TiO_2_-based photocatalysts [185]. The combination of TiO_2_ and Ag obtains results in visible light [186,187]. Ag-TiO_2_ NPs can act against *S. mutans* [186].

TiO_2_ can also have an anti-inflammatory action by (i) reducing platelet numbers; and (ii) increasing thrombin–antithrombin levels [63].

Moreover, TiO_2_ is an effective whitener used as a pigment in many medical and industrial products. Komatsu and colleagues proposed the use of TiO_2_ nanotubes in tooth whitening [188]. They demonstrated that the use of TiO_2_ nanotubes in addition to H_2_O_2_ at low concentrations enhanced whitening. The combination of TiO_2_ nanotubes with H_2_O_2_ increased the response to visible light [188].

Recent studies indicate that the use of TiO_2_ NPs can have toxic effects [189,190,191]. Moreover, Geraets and colleagues demonstrated that in rats, when oral exposure was repeated, TiO_2_ NPs can accumulate in tissue [192]. Rompelberg and colleagues, using analytic methods, concluded that toothpaste (in young children only) is the product that contributes most to TiO_2_ intake, but they did not draw a conclusion concerning the risk of general health [193]. In a review, Baranowska-Wójcik and colleagues explained that TiO_2_ NPs in small but regular doses can act on the intestinal mucosa, the heart, the brain, and other internal organs, which can increase the risk of developing systemic diseases or tumors [194].

#### 3.1.6. Copper Oxide Nanoparticles

Contrary to the metal oxides previously described, few studies exist concerning copper or copper oxide. A range of Cu and CuO NPs have been obtained from plant extracts such as magnolia, *Syzygium aromaticum*, *Euphorbia nivulia* [122], *Triticum aestivum* [195], *Fusarium solani* [196], and *Aloe vera* extract [197].

CuO NPs demonstrated anti-bacterial [197] and antifungal [196] activities (Table 1). An inverse relationship between the size of CuO NPs and the anti-bacterial activity has been established in vitro [197]. The greatest bactericidal activity was obtained for copper NPs ranging from 1 to 10 nm. CuO is cheaper than Ag, relatively stable in terms of both physical and chemical properties, and easily mixed with polymers. The physical and chemical characterization of CuO NPs revealed that CuO NPs synthetized by thermal plasma technology have sizes ranging from 20 to 95 nm and a mean surface area of 15.7 m^2^/g. In suspension, CuO NPs demonstrated their capacity to fight against a range of bacterial species. However, compared with silver, CuO NPs have lower bactericidal activity [198]. Cu_2_O NPs and CuO NPs have similar anti-bacterial activity [199]. CuO demonstrated a different anti-bacterial activity against multiple Gram-negative and Gram-positive species such as *P. aeruginosa*, methicillin-resistant *S. aureus* (MRSA), *Staphylococcus epidermidis*, and *E. coli* [199]. Amiri and colleagues demonstrated that CuO NPs had a high anti-microbial effect against the examined dental caries bacterial agents (*S. mutans*, *Lactobacillus acidophilus*, and *Lactobacillus casei*), and lower effect on three species of candida (*C. albicans*, *Candida krusei*, and *Candida glabrata*) [200].

#### 3.1.7. Other Nanoparticles Used in Oral Care Products and Their Anti-bacterial Activity

• Chlorhexidine NPs

In oral care products, one of the most common anti-microbial agents is CHX (Table 1) [201]. Recently, a novel approach, named nano-encapsulation, has emerged that delivers biologically potent compounds more effectively to specific targets [202]. Nano-encapsulated drugs can increase overall biological efficacy through rapid penetration and bioavailability while decreasing potential cytotoxicity, drug dosage, and the production costs, compared to controls [203].

Seneviratne and colleagues described a novel synthesis of mesoporous silica NPs encapsulated with pure (non-salt-form) CHX, namely CHX NPs [204]. They coated CHX on mesoporous silica NPs with inner pore channels of approximately 2.5 nm. The results obtained demonstrated that these CHX NPs had anti-bacterial effects against both planktonic and biofilm bacteria. Nano-CHX can act against several oral pathogens such as *A. actinomycetemcomitans*, *E. faecalis*, *F. nucleatum*, *S. mutans*, and *Streptococcus sobrinus* (*S. sobrinus*) in planktonic modes and in mono-species biofilms, respectively. Moreover, CHX NPs can inhibit the growth of multi-species oral biofilm composed of *S. sobrinus*, *P. gingivalis*, and *F. nucleatum* [204].

Barbour and colleagues developed novel anti-microbial NPs based on a hexametaphosphate salt of CHX [202]. These CHX NPs of size between 20 and 160 nm have colloidal structure and are highly negatively charged NPs (−50 mV). They are active against *MRSA* and *Pseudomonas aeruginosa* in both planktonic and biofilm conditions. Poly-lactic-co-glycolic acid NPs were also investigated as CHX biodegradable nanocarriers with gradual CHX release [3]. These new NPs could be added to toothpaste or mouthwash [202].

• Ferumoxytol

Ferumoxytol NPs can disrupt oral biofilms and avoid caries lesions via intrinsic peroxidase-like activity. Ferumoxytol NPs penetrate biofilm, bind to bacteria, and produce free radicals from hydrogen peroxide, provoking in situ death of bacteria because the cell membrane is disrupted, and the extracellular polymeric substance matrix is degraded. Combinate with low H_2_O_2_ concentrations, ferumoxytol inhibits biofilm accumulation on natural teeth and avoids acid damage to mineralized tissue in ex vivo biofilm models [205]. In vivo, in a rodent model with carious disease, topical oral treatment comprising ferumoxytol and H_2_O_2_ restrained the development of dental caries, avoiding the occurrence of severe tooth caries. Toothpaste and mouthwash containing ferumoxytol NPs could be an effective topical treatment of biofilm-induced oral disease [205].

• *Scutellaria baicalensis*

In traditional Chinese medicine, *Scutellaria baicalensis* is usually prescribed to treat infectious and inflammatory diseases [206]. It is used to manage periodontal disease [207]. Its anti-inflammatory, antioxidative, and immunomodulating action is due to the presence of baicalin, a flavonoid compound [208,209]. *Scutellaria baicalensis* has been loaded to magnetic NPs [91,210,211] and chitosan NPs [212]. These studies demonstrated that the loading of *Scutellaria baicalensis* on NPs increased the percentage of drug delivery.

Leung and colleagues analyzed the anti-bacterial activity of the mix of *Scutellaria baicalensis* NPs and CHX NPs (ratio 9:1 (w/w)) in both planktonic and biofilm bacteria. The mix was effective on mono-species biofilms of *A. actinomycetemcomitans*, *F. nucleatum*, *S. sobrinus*, and *S. mutans* at 24 h. It was also active against biofilms composed of multi-species such as *A. actinomycetemcomitans*, *P. gingivalis*, *F. nucleatum*, and *S. mutans* at 24 h. The author clearly showed a synergistic anti-bacterial action of mixed NPs on usual oral bacterial biofilms, which may be initiated as a novel anti-microbial factor for clinical oral care [213].

• *Chitosan nanoparticles*

Chitosan is an abundant polysaccharide produced by alkaline hydrolysis of chitin, the primary structural component of the crustaceans, arthropods shells, and fungal cell walls [214]. A large focus has fallen on this glucosamine unit-based linear biopolymer thanks to its unique cationic global charge, which no other polysaccharide has [215]. Chitosan showed both a high biocompatibility and anti-microbial activity against oral biofilms due in part to its positive charge, which facilitates its adhesion to bacteria cell walls, damaging the cell structure [216]. According to Costa and colleagues, chitosan proved its ability to destabilize the *S. mutans* and other oral microorganism (*P. intermedia*, *C. albicans*) biofilm formation and adherence more precisely when incorporated into a mouthwash [12,217].

Furthermore, chitosan NPs were tested as a low-cost ingredient of dental varnishes for their anti-microbial activity against *S. mutans* [11], and as a hybrid chitosan hydrogel for encapsulating Ag NPs to constitute an alternative for oral anti-microbial control (Table 1) [218]. Chitosan hydrogel was shown to have bactericide properties similar to chlorhexidine, and can release Ag NPs for two weeks. Moreover, this human RCT study proved that chitosan-containing polyherbal toothpaste significantly reduces plaque index and bacterial count [219]. Other studies demonstrated that chitosan toothpaste can act as an anti-erosive agent [220,221]. For example, Schueter and colleagues, in a human RCT study, demonstrated that the efficacy of tin or tin and fluoride chitosan toothpaste was of great interest in the case of erosion or hypersensitivity [221]. In another human RCT, Uysal and colleagues obtained the same type of results, and proved that toothpaste containing chitosan may reduce demineralization of enamel in patients presenting poor oral hygiene [222].

## 4. Nanoparticles as Remineralization Agents

To control the risk of caries and dental sensitivity of demineralized enamel, the action the remineralizing agents is required to restore structure and ensure enamel mechanical feature preservation. Indeed, the carious process corresponds to the demineralization of hard tissues in the tooth (enamel and dentine) due to attack from acids produced by bacteria from biofilms of dental plaque [223,224]. In the presence of fermentable carbohydrates, cariogenic bacteria grow and produce acid responsible for enamel demineralization [225,226]. To avoid caries, it is possible, as previously described, to fight against bacteria. However, another possibility is to favor the remineralization of tissues (Figure 1) [227]. Another major dental problem is dental erosion which corresponds to a progressive and irreversible loss of hard dental tissues due to a chemical process i.e., dissolution from acids not involving bacteria [228]. This can induce undesirable effects such as dental tissue loss, aesthetic defects, decreased vertical dimensions, and functional problems that may impact quality of life [228]. Moreover, the exposure of dentin, the internal tissue of the tooth, can lead to dental hypersensitivity. Hyperesthesia is related to the movement of fluids in dentinal tubules. It is characterized by an exaggerated reaction to a benign stimulus, unrelated to bacteria, and corresponds to a chronic pathology with acute episodes, specifically acute and brief pain [229].

Due to the fact that fluoride has demonstrated good properties of remineralization [230], few studies on NPs that have a remineralization effect have been undertaken. The main NPs known and used to remineralize are hydroxyapatite (HA) NPs (Table 1).

### 4.1. Hydroxyapatite

The major advantages of hydroxyapatite (HA) NPs are its resemblance to the mineral structure of teeth, bioactivity, and biocompatibility [231,232]. Indeed, particles are “similar in morphology and crystal structure to dental apatite” [233]. NPs with a size of 20 nm that correspond to characteristics of the natural building blocks of enamel had a strong affinity with demineralized surfaces [234,235]. Scanning electron microscopy analysis confirmed that these HA NPs are able to bind to pores created by demineralization [235]. In vitro, after adhering, HA NPs multiply and organize into microclusters to form an uniform apatite layer that completely overlaps interprismatic and prismatic enamel [235]. Similar results were observed by Roveri and colleagues with carbonated HA NPs with a size of 100 nm. HA NPs covered the demineralized enamel surfaces that fluoridated toothpaste more effectively [236]. Treatment with HA NPs exhibited a surface-to-Ca/P ratio similar to that of biological enamel and synthetic nanohydroxyapatite used, indicating an apatite coating deposition on the demineralized enamel surface [235]. Chen and colleagues described the mechanism of enamel prism formation with HA NPs [237]. The proteins of the enamel (mainly amelogenin and amelogenin fragments) change enamel crystals. Negatively charged carboxy-terminal hydrophilic parts of amelogenin protein bind to the charged surface of the enamel crystal. The surface of the crystal becomes hydrophobic either because the hydrophobic part of amelogenin is exposed or because the charged part of the nanospheres is digested. Thus, the hydrophobic HA NPs can gather together to form a thermodynamically stable prism structure such as the one observed in the enamel [237].

HA NPs have been integrated into products for oral care such as dentifrices and mouthwash to reduce or delete dental sensitivity by obstructing open dentinal tubules on the surface of the dentin and connected to the pulp, or for the purpose of promoting the remineralization of enamel by replacing calcium and phosphate ions in the areas from which minerals dissolved, restoring its integrity and shine [238,239,240,241]. In 2006, in Europe, HA NP dentifrice was instituted to facilitate remineralization and restoration of enamel [179]. In accordance with the producer, the maximal incorporation of this 15.5% HA NP aqueous suspension oral care ingredient is 20% in a dentifrice and 10% in a mouthwash [241].

Kani and colleagues were the first to prove, in one human RCT study, the advantage of using HA NPs toothpaste to remineralize [242]. They compared the use of 5% HA NP toothpaste with the use of the same toothpaste without HA NPs for 3 years. The caries incidence decreased by 56% in children brushing with a HA NP toothpaste compared to the group brushing with a control toothpaste [242]. Several in vitro studies reported the efficacity of HA NPs in the remineralizing process [240,243,244,245]. HA NP toothpaste as remineralizing paste showed better results when compared to toothpaste containing calcium and potassium ions and sodium nitrate [246]. In vitro, HA NPs appeared to be greater desensitizing agents than novamin and proargin, which are known to be desensitizing agents [247]. Moreover, several RCTs demonstrated the efficiency of HA NP toothpaste in reducing dentine hypersensitivity and stimulating a remineralizing mechanism [33,238,239,248,249,250,251]. Therefore, the decreasing of dentine hypersensitivity can be explained by the fact that the deposition of HA NPs produces a remineralizing effect on the enamel surface [236,252]. Bossù and colleagues confirmed, in vitro and in vivo, these results by demonstrating that HA NPs contained in toothpaste promote enamel regeneration with a biomimetic film similar in terms of morphology and structure to the biologic hydroxyapatite of the enamel [252]. A coating of a new layer of apatite is deposited, which contains fewer particles than the natural enamel. Moreover, due to the chemical bonds between the synthetic and natural crystals of the enamel, this new layer of apatite shows resistance to toothbrushing [252]. In one human RCT study, Browning and colleagues demonstrated that HA NPs can help to treat dental hypersensitivity after teeth-whitening. Indeed, for 14 days, subjects used a hydrogen peroxide gel and brushed their teeth with a dentifrice containing HA NPs. Fewer subjects using HA NP toothpaste suffered hypersensitivity than subjects who did not use the HA NP toothpaste and, and the hypersensitivity persisted for fewer days [253]. Another property of HA NP toothpaste is its positive effect on teeth-whitening and brightness [254,255].

Due to these properties of remineralization and desensitization, HA NPs could be an interesting alternative to fluoride toothpaste, which is recommended to prevent caries [256]. Its use led to a decrease in caries in industrialized countries [257], but the daily use of fluoride toothpaste is a potential source of fluorosis [258]. Moreover, Tschoope and colleagues compared fluoride toothpaste and HAP NP toothpaste, and demonstrated, in vitro, that the HAP NP toothpaste displayed higher remineralizing effects on both hard tissues than fluoride toothpaste [259]. The same observations were obtained by Manchery and colleagues [260]. The use of fluoride toothpaste does not impact the surface-area-to-Ca/P ratio following demineralization, contrary to the Ca/P ratio observed with the use of HA NP toothpaste, which is close to biological enamel [235]. Only modification of the structure of apatite is observed. A partial hydroxyl group is replaced by fluoride ions, whereas the calcium and phosphate content is not changed [235]. Hill and colleagues proved that use of toothpaste containing both HA NPs and fluoride had significant benefit in preventing demineralization and promoting the formation of fluoridated apatite, which is more resistant to acid attack [244]. Moreover, toothpaste containing HA and Zn NPs demonstrated a greater efficacy than fluoride toothpaste to prevent the demineralization of enamel induced by acidic products [261]. Due to the presence of Zn, this toothpaste also decreased the risk of gingivitis and periodontitis [262].

Mouthwash containing HA and Zn NPs have the same effect on enamel and prevent the multiplication of bacteria in the oral cavity [263,264]. The low number of studies concerning HA NP mouthwash have focused on its capacity to act on bacterial colonization. Kensche and colleagues demonstrated that HA NP mouthwash can help control bacterial biofilm formation. They observed, in situ, an accumulation of HA aggregates and salivary bacteria at the tooth surface. The number of bacteria that attach to the surface of the pellicle decreased compared with a control mouthwash and was similar compared with chlorhexidine 0.2% mouthwash [265]. The action of HA NPs is due more to anti-adhesive capacity than anti-bacterial effect. For one, the attachment of HA NPs to biofilm bacteria prevents the adhesion of new bacteria. Bacteria that adhere to HA NPs can also be eliminated by desorption of the complexes composed of HA NPs and the biofilm bacteria surface. For another, adhesins from the cell wall of saliva bacteria can be blocked by the accumulation of HA NPs, and bacteria could even be agglutinated by the interaction with the HA microclusters. Therefore, bacteria would not be able to adhere to the biofilm surface [265]. In vitro, Hannig and colleagues concluded that mouthwash containing HA and Zn NPs had anti-adherence properties and anti-bacterial effects against cariogenic bacteria such as *S. mutans* [263,266].

Despite the good performance of HA NPs, it is essential to ensure that it is not toxic for consumers. The Scientific Committee on Consumer Safety analyzed the safety of HA NPs as an oral care ingredient, but the results were not informative enough. Ramis and colleagues proved from in vitro experiments that no deleterious effects were noticed for human gingival epithelium tissues incubated with HA NPs at all evaluated time-points and parameters [241]. Moreover, after 7.5 min at 37 °C, the complete dissolution of 3.1% HA NPs in simulated gastric fluid was observed. These results confirmed the safety of HA NPs for oral care products [241].

### 4.2. Casein Phosphopeptide–Amorphous Calcium Phosphate

Calcium phosphate particles dissolved in casein milk protein is a new remineralization method that creates, in the acidic environment of dental plaque, an amorphous calcium phosphate particle super-saturation around the tooth that increases the remineralization process [267]. Indeed, the casein phosphopeptides can stabilize calcium phosphate in solution. The calcium phosphates bind with phosphoserine residues to form casein phosphopeptide–amorphous calcium phosphate (CPP–ACP) clusters [268]. These CPP–ACP nanocomplexes are able to localize at the surface of the tooth, bringing about buffering of the phosphate and calcium free ion activities, and then help maintain a state of super-saturation with respect to tooth enamel, negating demineralization and enhancing remineralization [269,270]. Indeed, these CPP–ACP nanocomplexes create a calcium and phosphate reservoir that can bind to plaque and dental surfaces [268].

Rao and colleagues undertook a clinical trial to assess the efficacy of toothpaste containing CPP in preventing carious lesions [271]. For 24 months, 150 schoolchildren brushed their teeth with a given toothpaste. Fifty used toothpaste containing CPP (2% *w*/*w*), 50 used toothpaste containing fluoride (1190 mg/kg) as sodium monofluorophosphate (0.76%), and 50 used a placebo toothpaste without CPP or fluoride. The conclusions of the study were that CPP can be incorporated into calcium carbonate-based toothpaste, and that toothpaste containing CPP can help prevent carious lesions. More recently, several in vitro and in situ studies have demonstrated that toothpaste containing CPP–ACP nanocomplexes could avoid enamel demineralization produced by soft drinks [272,273,274]. Moreover, in case of bleaching protocols, this type of toothpaste can prevent negative changes of roughness and hardness to enamel [275]. Therefore recently CPP–ACP and fluoride has been recommended as treatment to remineralize initial caries and white spot lesions [276,277,278,279]. However, CPP–ACP compared to fluoride has a slightly lower potential in the remineralization of early enamel caries [276,277,278,279]. An in vitro study concluded that toothpaste containing CPP–ACP NPs with *L. rhamnosus* (probiotic strain) had a potential remineralizing property with a more promising anti-microbial efficiency [280].

### 4.3. Bioactive Glass

Several bioactive glasses (BAG) exist, such as phosphate-based glass and silicate-based glass. BAG is known to heal bone defects and stimulate bone regeneration [281]. Similar action is observed on the tooth. Indeed, BAG NPs can decrease tooth sensitivity by mineralizing dentine tubules. First, the BAG makes contact with an aqueous solution, and the NPs will change to a mesoporous shape. This layer of NPs allows formation of apatite on the dentine surface. Then, a pH rise provokes the precipitation of HA. The mineralizing process can be activated by phosphate and calcium ions contained in the bioactive glass and mineralizing agents from the saliva [282]. Due to larger surface areas and higher Ca/P ratios, better remineralization and slower progression of carious lesions are observed for BAG NPs compared to conventional BAGs [283,284,285].

Due to this remineralizing property, BAG NPs are incorporated into toothpaste to treat dentine hypersensitivity [286,287,288,289]. In vitro studies have demonstrated that BAG NPs could promote mineral formation on dentin surfaces and they were shown to make dentin more acid-resistant [290,291]. Calcium sodium-phosphosilicate toothpaste was more effective in increasing acid resistance compared with CCP–ACP NP toothpaste [291]. Rajendran and colleagues showed, in vitro, that toothpaste containing BAG NPs (calcium sodium-phosphosilicate) was less effective than toothpaste containing CPP–ACP NPs to remineralize the enamel of carious lesions [292]. Even if the new HA layer formed in response to the presence of BAG NPs is similar to those of enamel or dentine, it presents better resistance to abrasion [293]. In vitro, Farooq and colleagues proved that fluoride-containing bioactive glass had better capacities of remineralization compared to BAG toothpaste and sodium monofluorophosphate toothpaste [294]. In a randomized clinical trial, Patel and colleagues demonstrated that the 5% fluorocalcium phosphosilicate-containing bioactive glass toothpaste further reduced hypersensitivity compared with an 8% arginine and calcium carbonate toothpaste and a placebo toothpaste [295].

Moreover, BAG NPs toothpaste has demonstrated anti-bacterial properties [281]. In their systematic review, Dai and colleagues explained that very few and only in vitro studies investigated the anti-microbial effect of BAG NPs because researchers focused on the remineralizing capacities [281]. However, a concentration of BAG NPs twice that of CMB can inhibit *S. mutans* biofilm [296]. To fight bacteria, BAG NPs release alkaline ions that cause the pH to rise and thus create an environment in which bacteria cannot grow. 

A higher resistance to acid dissolution is obtained by adding fluoride to BAG, allowing formation of fluorapatite on the tooth surface [297]. This deposit of fluoroapatite on the dentine surface occludes dentine tubules and decreases permeability [298]. Strontium can also be added to BAG to increase bonding capacities [299]. The dissolution of hydroxyapatite by the acid produced by cariogenic bacteria can be inhibited by the incorporation of strontium and fluoride into BAG. Strontium can replace calcium for precipitate formation, and it has synergistic caries inhibition effect with fluoride. Therefore, due to its capacities to remineralize dental hard tissues, strontium can prevent caries lesions [300].

## 5. Complementary Content

Cosmetic products are placed in contact with external parts of the human body, with mucous membranes of the oral cavity, and with teeth. The development of nanotechnology has opened up new perspectives for innovation in oral cosmetics; simultaneously, the use of very small particles in user products has increased preoccupation about their safety to human health [301,302]. Due to the potential for modifications related to biokinetic behavior, toxicological effects, and the size of the physico-chemical properties of materials at the nanometric scale, exposure to NPs through the use of cosmetic products can constitute a risk of harmful consequences from persistent and insoluble nanoparticles that can arrive at involuntary sites in the organism and interact with biological entities close to molecular proportions [303].

In the case of NPs, the chemical composition is not alone in influencing potential toxicity. Other properties, including size, play a role in this, allowing NPs to escape the body’s defense mechanisms and to interact with biological constituents [38]. Consequently, the safety-in-use of these produces has been established by measuring the substances, their toxicity profiles, chemical structures, and exposure types [303,304]. Specific attention is required for long-term safety considerations, as cosmetic products can be widely used over a large part of the human lifespan and sensitive groups of the population, such as children, may be affected [303].

In practice, cosmetic products have seldom been related to severe health hazards, which, however, does not imply that cosmetics are secure in use per se (SCCS 8th revision). However, among all cosmetic products, taking an interest in the risks linked to oral exposure to oral cosmetics such as toothpaste and mouthwash is relevant because they can be inadvertently ingested. If, to assess oral exposure, the procedure followed is similar to that used for NPs for other cosmetic ingredients [305], it is considered that the size and agglomeration state of NPs may differ from having low pH in the stomach and high ionic strength throughout the gastrointestinal tract. NPs can even lose their nano-specific properties, due to a deterioration or dissolution. For such NPs, properties and effects are more probably similar to those of the corresponding ions [306,307].

Estimated daily exposure levels for toothpaste in adult and mouthwash cosmetic product types according to Cosmetics Europe data were described in detail by Hall and colleagues [308]. A literature overview (2016–2018) of specific cosmetic consumers described exposure data and assessments by product category, of which three concerned toothpaste [303]. Gomez-Berrada and colleagues evaluated in French families the consumption and exposure to dentifrice, leaving consumers free to use their own product at home in accordance with their practices [309]. In 2018, Bernard and colleagues produced a probabilistic evaluation of the exposure of oral cosmetics using recent data from French consumption across different age groups [310]. Finally, Strittholt and colleagues, in an RCT study, looked for whether there was a difference in the amount of toothpaste ingested by children using pea-size dosing instructions [311].

## 6. Conclusions

Nanotechnology has brought enormous changes to the field of preventive dentistry. Currently, these developments are found on a large scale in various activities related to oral prophylaxis. Currently, oral care products such as toothpastes and mouthwash contain NPs with anti-microbial, anti-inflammatory, and remineralizing properties. Because of promising results and varied and often unpublished properties, nanomaterials contain very diverse potentialities, and their uses prompt multiple perspectives that make it possible for them to be effective. However, the advantages of NPs are the same reasons that make them dangerous—small size, surface properties, quantum state, migration, aggregation, mutation, and the creation of free radicals. Therefore, research into NPs is currently one of the most studied branches of science, due to the almost unlimited fields of application, and therefore regulatory and safety concerns must be discussed and questioned, especially concerning the use of hydroxyapatite NPs in oral care products.

## Figures and Tables

**Table 1:** Classification of nanoparticles contained in oral care products according to their action.

	Nanoparticles	
Anti-microbial	Anti-inflammatory	Remineralizing
Oxide Silver Gold	Oxide Silver Gold	Hydroxyapatite Casein phosphopeptide–amorphous Calcium phosphate Tin chitosan Tin/Fluor chitosan Bioactive glass
Metal oxide Zinc oxide Titanium dioxide Copper oxide	Metal oxide Zinc oxide Titanium dioxide	
Chlorhexidine Ferumoxytol *Scutellaria baicalensis* Chitosan Bioactive glass		

## References

[1] Lövestam G., Rauscher H., Roebben G., Klüttgen B.S., Gibson N., Putaud J.-P., Stamm H. (2010). Considerations on a definition of nanomaterial for regulatory purposes. JCR Ref. Rep. Luxemb..

[2] Boulaiz H., Alvarez P.J., Ramirez A., Marchal J.A., Prados J., Rodríguez-Serrano F., Perán M., Melguizo C., Aranega A. (2011). Nanomedicine: Application Areas and Development Prospects. Int. J. Mol. Sci..

[3] Priyadarsini S., Mukherjee S., Mishra M. (2018). Nanoparticles used in dentistry: A review. J. Oral Biol. Craniofacial Res..

[4] Hassan L. (2017). Dental Medicine Nanosystems: Nanoparticles and their use in Dentistry and Oral Health Care. Int. J. Dent. Oral Health.

[5] Smith R. (2015). Regulation (EC) No 764/2008 of the European Parliament and of the Council. Core EU Legislation.

[6] Global $36+ Billion Toothpaste Market, 2024: Growth, Trends and Forecast Analysis from 2019—ResearchAndMarkets.com. https://www.businesswire.com/news/home/20190509005698/en/Global-36-Billion-Toothpaste-Market-2024-Growth.

[7] Creusen M.E.H., Schoormans J.P.L. (2005). The Different Roles of Product Appearance in Consumer Choice *. J. Prod. Innov. Manag..

[8] Catalogue of nanomaterials in cosmetic products placed on the market—European Commission. https://www.endseurope.com/article/1666277?utm_source=website&utm_medium=social.

[9] Jeevanandam J., Barhoum A., Chan Y.S., Dufresne A., Danquah M.K. (2018). Review on nanoparticles and nanostructured materials: History, sources, toxicity and regulations. Beilstein J. Nanotechnol..

[10] Corrie S.R., Thurecht K.J. (2016). Nano-Bio Interactions: Guiding the Development of Nanoparticle Therapeutics, Diagnostics, and Imaging Agents. Pharm. Res..

[11] Wassel M.O., Khattab M.A. (2017). Antibacterial activity against Streptococcus mutans and inhibition of bacterial induced enamel demineralization of propolis, miswak, and chitosan nanoparticles based dental varnishes. J. Adv. Res..

[12] Costa E.M., Silva S., Madureira A.R., Cardelle-Cobas A., Tavaria F.K., Pintado M.M. (2014). A comprehensive study into the impact of a chitosan mouthwash upon oral microorganism’s biofilm formation in vitro. Carbohydr. Polym..

[13] Cadinoiu A., Darabă O., Merlușcă P., Anastasiu D., Vasiliu M., Chirap A., Bîrgăoanu A., Burlui V. (2014). Liposomal formulations with potential dental applications. Int. J. Med. Dent..

[14] Sato K. (2007). Mechanism of Hydroxyapatite Mineralization in Biological Systems (Review). J. Ceram. Soc. Jpn..

[15] Mitrano D.M., Motellier S., Clavaguera S., Nowack B. (2015). Review of nanomaterial aging and transformations through the life cycle of nano-enhanced products. Environ. Int..

[16] Ashraf M.A., Peng W., Zare Y., Rhee K.Y. (2018). Effects of Size and Aggregation/Agglomeration of Nanoparticles on the Interfacial/Interphase Properties and Tensile Strength of Polymer Nanocomposites. Nanoscale Res. Lett..

[17] Ball R.L., Bajaj P., Whitehead K.A. (2017). Achieving long-term stability of lipid nanoparticles: Examining the effect of pH, temperature, and lyophilization. Int. J. Nanomed..

[18] Ma X., Zare Y., Rhee K.Y. (2017). A Two-Step Methodology to Study the Influence of Aggregation/Agglomeration of Nanoparticles on Young’s Modulus of Polymer Nanocomposites. Nanoscale Res. Lett..

[19] Kah J.C.Y. (2013). Stability and aggregation assays of nanoparticles in biological media. Nanomaterial Interfaces in Biology.

[20] Veilleux D., Nelea M., Biniecki K., Lavertu M., Buschmann M.D. (2016). Preparation of Concentrated Chitosan/DNA Nanoparticle Formulations by Lyophilization for Gene Delivery at Clinically Relevant Dosages. J. Pharm. Sci..

[21] Liu H., Zhang G., Lu L., Chen Y., Luo M., Bian J., Wang Z., Wang L. Influence of Varied Fluorine Contents on Long-Term Storage Stability of Polyacrylate Nanoparticles and Film Properties. https://www.hindawi.com/journals/jnm/2019/2970819/.

[22] Ristroph K.D., Feng J., McManus S.A., Zhang Y., Gong K., Ramachandruni H., White C.E., Prud’homme R.K. (2019). Spray drying OZ439 nanoparticles to form stable, water-dispersible powders for oral malaria therapy. J. Transl. Med..

[23] Ngamcherdtrakul W., Sangvanich T., Reda M., Gu S., Bejan D., Yantasee W. (2018). Lyophilization and stability of antibody-conjugated mesoporous silica nanoparticle with cationic polymer and PEG for siRNA delivery. Int. J. Nanomed..

[24] Howard M.D., Lu X., Jay M., Dziubla T.D. (2012). Optimization of the lyophilization process for long-term stability of solid-lipid nanoparticles. Drug Dev. Ind. Pharm..

[25] Fonte P., Reis S., Sarmento B. (2016). Facts and evidences on the lyophilization of polymeric nanoparticles for drug delivery. J. Control. Release.

[26] Abdelwahed W., Degobert G., Stainmesse S., Fessi H. (2006). Freeze-drying of nanoparticles: Formulation, process and storage considerations. Adv. Drug Deliv. Rev..

[27] Abiodun-Solanke I., Ajayi D., Arigbede A. (2014). Nanotechnology and its Application in Dentistry. Ann. Med. Health Sci. Res..

[28] Khan S., Al-Khedhairy A., Musarrat J. (2015). ZnO and TiO_2_ nanoparticles as novel antimicrobial agents for oral hygiene: A review. J. Nanoparticle Res..

[29] Huh A.J., Kwon Y.J. (2011). “Nanoantibiotics”: A new paradigm for treating infectious diseases using nanomaterials in the antibiotics resistant era. J. Control. Release.

[30] Hussein-Al-Ali S.H., El Zowalaty M.E., Hussein M.Z., Geilich B.M., Webster T.J. (2014). Synthesis, characterization, and antimicrobial activity of an ampicillin-conjugated magnetic nanoantibiotic for medical applications. Int. J. Nanomed..

[31] Ahmed F., Prashanth S.T., Sindhu K., Nayak A., Chaturvedi S. (2019). Antimicrobial efficacy of nanosilver and chitosan against Streptococcus mutans, as an ingredient of toothpaste formulation: An in vitro study. J. Indian Soc. Pedod. Prev. Dent..

[32] Alsubaie A.A., Sarfraz Z., Alali A.A., Alessa A.E., Subaie H.A.A., Shah A.T., Khan A.S. (2019). Effect of nano-zinc oxide and fluoride-doped bioactive glass-based dentifrices on esthetic restorations. Dent. Med. Probl..

[33] Vano M., Derchi G., Barone A., Genovesi A., Covani U. (2015). Tooth bleaching with hydrogen peroxide and nano-hydroxyapatite: A 9-month follow-up randomized clinical trial. Int. J. Dent. Hyg..

[34] Coelho C.C., Grenho L., Gomes P.S., Quadros P.A., Fernandes M.H. (2019). Nano-hydroxyapatite in oral care cosmetics: Characterization and cytotoxicity assessment. Sci. Rep..

[35] Frencken J.E., Sharma P., Stenhouse L., Green D., Laverty D., Dietrich T. (2017). Global epidemiology of dental caries and severe periodontitis—A comprehensive review. J. Clin. Periodontol..

[36] Valm A.M. (2019). The Structure of Dental Plaque Microbial Communities in the Transition from Health to Dental Caries and Periodontal Disease. J. Mol. Biol..

[37] Sanz M., Beighton D., Curtis M.A., Cury J.A., Dige I., Dommisch H., Ellwood R., Giacaman R.A., Herrera D., Herzberg M.C. (2017). Role of microbial biofilms in the maintenance of oral health and in the development of dental caries and periodontal diseases. Consensus report of group 1 of the Joint EFP/ORCA workshop on the boundaries between caries and periodontal disease. J. Clin. Periodontol..

[38] Wang L., Hu C., Shao L. (2017). The antimicrobial activity of nanoparticles: Present situation and prospects for the future. Int. J. Nanomed..

[39] Mah T.-F. (2012). Biofilm-specific antibiotic resistance. Future Microbiol..

[40] Reyes L., Herrera D., Kozarov E., Roldán S., Progulske-Fox A. (2013). Periodontal bacterial invasion and infection: Contribution to atherosclerotic pathology. J. Clin. Periodontol..

[41] Seil J.T., Webster T.J. (2012). Antimicrobial applications of nanotechnology: Methods and literature. Int. J. Nanomed..

[42] Malarkodi C., Rajeshkumar S., Paulkumar K., Vanaja M., Gnanajobitha G., Annadurai G. (2014). Biosynthesis and Antimicrobial Activity of Semiconductor Nanoparticles against Oral Pathogens. Bioinorg. Chem. Appl..

[43] Armentano I., Arciola C.R., Fortunati E., Ferrari D., Mattioli S., Amoroso C.F., Rizzo J., Kenny J.M., Imbriani M., Visai L. (2014). The interaction of bacteria with engineered nanostructured polymeric materials: A review. Sci. World J..

[44] Li H., Chen Q., Zhao J., Urmila K. (2015). Enhancing the antimicrobial activity of natural extraction using the synthetic ultrasmall metal nanoparticles. Sci. Rep..

[45] Luan B., Huynh T., Zhou R. (2016). Complete wetting of graphene by biological lipids. Nanoscale.

[46] Gao W., Thamphiwatana S., Angsantikul P., Zhang L. (2014). Nanoparticle approaches against bacterial infections. Wiley Interdiscip. Rev. Nanomed. Nanobiotechnol..

[47] Yang W., Shen C., Ji Q., An H., Wang J., Liu Q., Zhang Z. (2009). Food storage material silver nanoparticles interfere with DNA replication fidelity and bind with DNA. Nanotechnology.

[48] Xu Y., Wei M.-T., Ou-Yang H.D., Walker S.G., Wang H.Z., Gordon C.R., Guterman S., Zawacki E., Applebaum E., Brink P.R. (2016). Exposure to TiO_2_ nanoparticles increases Staphylococcus aureus infection of HeLa cells. J. Nanobiotechnol..

[49] Zakharova O.V., Godymchuk A.Y., Gusev A.A., Gulchenko S.I., Vasyukova I.A., Kuznetsov D.V. (2015). Considerable Variation of Antibacterial Activity of Cu Nanoparticles Suspensions Depending on the Storage Time, Dispersive Medium, and Particle Sizes. BioMed Res. Int..

[50] Gurunathan S., Han J.W., Dayem A.A., Eppakayala V., Kim J.-H. (2012). Oxidative stress-mediated antibacterial activity of graphene oxide and reduced graphene oxide in Pseudomonas aeruginosa. Int. J. Nanomed..

[51] Leung Y.H., Ng A.M.C., Xu X., Shen Z., Gethings L.A., Wong M.T., Chan C.M.N., Guo M.Y., Ng Y.H., Djurišić A.B. (2014). Mechanisms of antibacterial activity of MgO: Non-ROS mediated toxicity of MgO nanoparticles towards Escherichia coli. Small Weinh. Bergstr. Ger..

[52] Dröge W. (2002). Free radicals in the physiological control of cell function. Physiol. Rev..

[53] Shaikh S., Nazam N., Rizvi S.M.D., Ahmad K., Baig M.H., Lee E.J., Choi I. (2019). Mechanistic Insights into the Antimicrobial Actions of Metallic Nanoparticles and Their Implications for Multidrug Resistance. Int. J. Mol. Sci..

[54] Nathan C., Cunningham-Bussel A. (2013). Beyond oxidative stress: An immunologist’s guide to reactive oxygen species. Nat. Rev. Immunol..

[55] Gold K., Slay B., Knackstedt M., Gaharwar A.K. (2018). Antimicrobial Activity of Metal and Metal-Oxide Based Nanoparticles. Adv. Ther..

[56] Cheloni G., Marti E., Slaveykova V.I. (2016). Interactive effects of copper oxide nanoparticles and light to green alga *Chlamydomonas reinhardtii*. Aquat. Toxicol. Amst. Neth..

[57] Ansari M.A., Khan H.M., Alzohairy M.A., Jalal M., Ali S.G., Pal R., Musarrat J. (2015). Green synthesis of Al_2_O_3_ nanoparticles and their bactericidal potential against clinical isolates of multi-drug resistant Pseudomonas aeruginosa. World J. Microbiol. Biotechnol..

[58] Castellano J.J., Shafii S.M., Ko F., Donate G., Wright T.E., Mannari R.J., Payne W.G., Smith D.J., Robson M.C. (2007). Comparative evaluation of silver-containing antimicrobial dressings and drugs. Int. Wound J..

[59] Polívková M., Štrublová V., Hubáček T., Rimpelová S., Švorčík V., Siegel J. (2017). Surface characterization and antibacterial response of silver nanowire arrays supported on laser-treated polyethylene naphthalate. Mater. Sci. Eng. C Mater. Biol. Appl..

[60] Zhang H., Lv X., Li Y., Wang Y., Li J. (2010). P25-graphene composite as a high performance photocatalyst. ACS Nano.

[61] Yu J., Zhang W., Li Y., Wang G., Yang L., Jin J., Chen Q., Huang M. (2014). Synthesis, characterization, antimicrobial activity and mechanism of a novel hydroxyapatite whisker/nano zinc oxide biomaterial. Biomed. Mater. Bristol Engl..

[62] Levard C., Hotze E.M., Lowry G.V., Brown G.E. (2012). Environmental Transformations of Silver Nanoparticles: Impact on Stability and Toxicity. Environ. Sci. Technol..

[63] Agarwal H., Nakara A., Shanmugam V.K. (2019). Anti-inflammatory mechanism of various metal and metal oxide nanoparticles synthesized using plant extracts: A review. Biomed. Pharmacother. Biomed. Pharmacother..

[64] Simko M., Fiedeler U., Gazsó A., Nentwich M. (2011). The impact of nanoparticles on cellular functions. Httphwoeawacatnanotrust-Doss.

[65] Kuhn D.A., Vanhecke D., Michen B., Blank F., Gehr P., Petri-Fink A., Rothen-Rutishauser B. (2014). Different endocytotic uptake mechanisms for nanoparticles in epithelial cells and macrophages. Beilstein J. Nanotechnol..

[66] Mahmoudi M., Lynch I., Ejtehadi M.R., Monopoli M.P., Bombelli F.B., Laurent S. (2011). Protein-nanoparticle interactions: Opportunities and challenges. Chem. Rev..

[67] Muñoz L.E., Bilyy R., Biermann M.H.C., Kienhöfer D., Maueröder C., Hahn J., Brauner J.M., Weidner D., Chen J., Scharin-Mehlmann M. (2016). Nanoparticles size-dependently initiate self-limiting NETosis-driven inflammation. Proc. Natl. Acad. Sci. USA.

[68] Lee S.H., Jun B.-H. (2019). Silver Nanoparticles: Synthesis and Application for Nanomedicine. Int. J. Mol. Sci..

[69] Beyene H.D., Werkneh A.A., Bezabh H.K., Ambaye T.G. (2017). Synthesis paradigm and applications of silver nanoparticles (AgNPs), a review. Sustain. Mater. Technol..

[70] Majoumouo M.S., Sibuyi N.R.S., Tincho M.B., Mbekou M., Boyom F.F., Meyer M. (2019). Enhanced Anti-Bacterial Activity Of Biogenic Silver Nanoparticles Synthesized From Terminalia mantaly Extracts. Int. J. Nanomed..

[71] Bansal V., Ramanathan R., Bhargava S.K. (2011). Fungus-mediated Biological Approaches Towards ‘Green’ Synthesis of Oxide Nanomaterials *. Aust. J. Chem..

[72] Gahlawat G., Choudhury A.R. (2019). A review on the biosynthesis of metal and metal salt nanoparticles by microbes. RSC Adv..

[73] Ali J., Ali N., Wang L., Waseem H., Pan G. (2019). Revisiting the mechanistic pathways for bacterial mediated synthesis of noble metal nanoparticles. J. Microbiol. Methods.

[74] Guilger-Casagrande M., de Lima R. (2019). Synthesis of Silver Nanoparticles Mediated by Fungi: A Review. Front. Bioeng. Biotechnol..

[75] Khan M., Shaik M.R., Adil S.F., Khan S.T., Al-Warthan A., Siddiqui M.R.H., Tahir M.N., Tremel W. (2018). Plant extracts as green reductants for the synthesis of silver nanoparticles: Lessons from chemical synthesis. Dalton Trans..

[76] Valsalam S., Agastian P., Esmail G.A., Ghilan A.-K.M., Al-Dhabi N.A., Arasu M.V. (2019). Biosynthesis of silver and gold nanoparticles using Musa acuminata colla flower and its pharmaceutical activity against bacteria and anticancer efficacy. J. Photochem. Photobiol. B.

[77] Das G., Patra J.K., Basavegowda N., Vishnuprasad C.N., Shin H.-S. (2019). Comparative study on antidiabetic, cytotoxicity, antioxidant and antibacterial properties of biosynthesized silver nanoparticles using outer peels of two varieties of *Ipomoea batatas* (L.) Lam. Int. J. Nanomed..

[78] Arya A., Mishra V., Chundawat T.S. (2019). Green synthesis of silver nanoparticles from green algae (*Botryococcus braunii*) and its catalytic behavior for the synthesis of benzimidazoles. Chem. Data Collect..

[79] Ahamed M., Alsalhi M.S., Siddiqui M.K.J. (2010). Silver nanoparticle applications and human health. Clin. Chim. Acta Int. J. Clin. Chem..

[80] Wright J.B., Lam K., Hansen D., Burrell R.E. (1999). Efficacy of topical silver against fungal burn wound pathogens. Am. J. Infect. Control.

[81] Wijnhoven S.W.P., Peijnenburg W.J.G.M., Herberts C.A., Hagens W.I., Oomen A.G., Heugens E.H.W., Roszek B., Bisschops J., Gosens I., Meent D.V.D. (2009). Nano-silver—A review of available data and knowledge gaps in human and environmental risk assessment. Nanotoxicology.

[82] Gurunathan S., Han J.W., Kwon D.-N., Kim J.-H. (2014). Enhanced antibacterial and anti-biofilm activities of silver nanoparticles against Gram-negative and Gram-positive bacteria. Nanoscale Res. Lett..

[83] Niakan S., Niakan M., Hesaraki S., Nejadmoghaddam M.R., Moradi M., Hanafiabdar M., Allamezadeh R., Sabouri M. (2013). Comparison of the Antibacterial Effects of Nanosilver With 18 Antibiotics on Multidrug Resistance Clinical Isolates of *Acinetobacter baumannii*. Jundishapur J. Microbiol..

[84] Li W.-R., Xie X.-B., Shi Q.-S., Zeng H.-Y., Ou-Yang Y.-S., Chen Y.-B. (2010). Antibacterial activity and mechanism of silver nanoparticles on Escherichia coli. Appl. Microbiol. Biotechnol..

[85] Niakan M., Azimi H.R., Jafarian Z., Mohammadtaghi G., Niakan S., Mostafavizade S.M. (2013). Evaluation of Nanosilver Solution Stability against *Streptococcus mutans*, *Staphylococcus aureus* and *Pseudomonas aeruginosa*. Jundishapur J. Microbiol..

[86] Sotoodehnia P., Mazlan N., Mohd Saud H., Samsuri W.A., Habib S.H., Soltangheisi A. (2019). Minimum inhibitory concentration of nano-silver bactericides for beneficial microbes and its effect on Ralstonia solanacearum and seed germination of Japanese Cucumber (Cucumis sativus). PeerJ.

[87] Pulit-Prociak J., Banach M. (2016). Silver nanoparticles—A material of the future…?. Open Chem..

[88] Lotfi M., Vosoughhosseini S., Ranjkesh B., Khani S., Saghiri M., Zand V. (2011). Antimicrobial efficacy of nanosilver, sodium hypochlorite and chlorhexidine gluconate against *Enterococcus faecalis*. Afr. J. Biotechnol..

[89] Zarei M., Jamnejad A., Khajehali E. (2014). Antibacterial effect of silver nanoparticles against four foodborne pathogens. Jundishapur J. Microbiol..

[90] Banach M., Tymczyna L., Chmielowiec-Korzeniowska A., Pulit-Prociak J. (2016). Nanosilver Biocidal Properties and Their Application in Disinfection of Hatchers in Poultry Processing Plants. Bioinorg. Chem. Appl..

[91] Cheng L., Zhang K., Weir M.D., Liu H., Zhou X., Xu H.H.K. (2013). Effects of antibacterial primers with quaternary ammonium and nano-silver on Streptococcus mutans impregnated in human dentin blocks. Dent. Mater..

[92] Panacek A., Kvítek L., Prucek R., Kolar M., Vecerova R., Pizúrova N., Sharma V.K., Nevecna T., Zboril R. (2006). Silver colloid nanoparticles: Synthesis, characterization, and their antibacterial activity. J. Phys. Chem. B.

[93] Morones J.R., Elechiguerra J.L., Camacho A., Holt K., Kouri J.B., Ramírez J.T., Yacaman M.J. (2005). The bactericidal effect of silver nanoparticles. Nanotechnology.

[94] Qasim M., Udomluck N., Chang J., Park H., Kim K. (2018). Antimicrobial activity of silver nanoparticles encapsulated in poly-N-isopropylacrylamide-based polymeric nanoparticles. Int. J. Nanomed..

[95] Li J., Rong K., Zhao H., Li F., Lu Z., Chen R. (2013). Highly selective antibacterial activities of silver nanoparticles against Bacillus subtilis. J. Nanosci. Nanotechnol..

[96] Qais F.A., Shafiq A., Khan H.M., Husain F.M., Khan R.A., Alenazi B., Alsalme A., Ahmad I. (2019). Antibacterial Effect of Silver Nanoparticles Synthesized Using *Murraya koenigii* (L.) against Multidrug-Resistant Pathogens. Bioinorg. Chem. Appl..

[97] Lok C.-N., Ho C.-M., Chen R., He Q.-Y., Yu W.-Y., Sun H., Tam P.K.-H., Chiu J.-F., Che C.-M. (2007). Silver nanoparticles: Partial oxidation and antibacterial activities. J. Biol. Inorg. Chem. JBIC Publ. Soc. Biol. Inorg. Chem..

[98] Sotiriou G.A., Pratsinis S.E. (2010). Antibacterial activity of nanosilver ions and particles. Environ. Sci. Technol..

[99] Nateghi M.R., Hajimirzababa H. (2014). Effect of silver nanoparticles morphologies on antimicrobial properties of cotton fabrics. J. Text. Inst..

[100] Lkhagvajav N., Koizhaiganova M., Yasa I., Çelik E., Sari Ö. (2015). Characterization and antimicrobial performance of nano silver coatings on leather materials. Braz. J. Microbiol..

[101] Ayala-Núñez N.V., Lara Villegas H.H., del Carmen Ixtepan Turrent L., Rodríguez Padilla C. (2009). Silver Nanoparticles Toxicity and Bactericidal Effect Against Methicillin-Resistant Staphylococcus aureus: Nanoscale Does Matter. NanoBiotechnology.

[102] Masri A., Anwar A., Khan N.A., Shahbaz M.S., Khan K.M., Shahabuddin S., Siddiqui R. (2019). Antibacterial Effects of Quinazolin-4(3H)-One Functionalized-Conjugated Silver Nanoparticles. Antibiotics.

[103] Elechiguerra J.L., Burt J.L., Morones J.R., Camacho-Bragado A., Gao X., Lara H.H., Yacaman M.J. (2005). Interaction of silver nanoparticles with HIV-1. J. Nanobiotechnol..

[104] Gaikwad S., Ingle A., Gade A., Rai M., Falanga A., Incoronato N., Russo L., Galdiero S., Galdiero M. (2013). Antiviral activity of mycosynthesized silver nanoparticles against herpes simplex virus and human parainfluenza virus type 3. Int. J. Nanomed..

[105] Mori Y., Ono T., Miyahira Y., Nguyen V.Q., Matsui T., Ishihara M. (2013). Antiviral activity of silver nanoparticle/chitosan composites against H1N1 influenza A virus. Nanoscale Res. Lett..

[106] Prabhu S., Poulose E.K. (2012). Silver nanoparticles: Mechanism of antimicrobial action, synthesis, medical applications, and toxicity effects. Int. Nano Lett..

[107] Simón-Soro A., Mira A. (2015). Solving the etiology of dental caries. Trends Microbiol..

[108] Junevičius J., Žilinskas J., Česaitis K., Česaitienė G., Gleiznys D., Maželienė Ž. (2015). Antimicrobial activity of silver and gold in toothpastes: A comparative analysis. Stomatologija.

[109] Balagopal S., Arjunkumar R. (2013). Chlorhexidine: The Gold Standard Antiplaque Agent. J. Pharm. Sci..

[110] Charles C.H., Mostler K.M., Bartels L.L., Mankodi S.M. (2004). Comparative antiplaque and antigingivitis effectiveness of a chlorhexidine and an essential oil mouthrinse: 6-month clinical trial. J. Clin. Periodontol..

[111] Esfahanian V., Mohamadi F., Amini S. (2012). An In Vitro Comparison of Antimicrobial Effect of Nanosil and ChlorhexidineMouthrinses. J. Islam. Dent. Assoc. Iran.

[112] Abu-Elteen K.H., Abu-Alteen R.M. (1998). The prevalence of Candida albicans populations in the mouths of complete denture wearers. New Microbiol..

[113] Belazi M., Velegraki A., Koussidou-Eremondi T., Andreadis D., Hini S., Arsenis G., Eliopoulou C., Destouni E., Antoniades D. (2004). Oral Candida isolates in patients undergoing radiotherapy for head and neck cancer: Prevalence, azole susceptibility profiles and response to antifungal treatment. Oral Microbiol. Immunol..

[114] Abadi M.F.D., Mehrabian S., Asghari B., Namvar A.E., Ezzatifar F., Lari A.R. (2013). Silver nanoparticles as active ingredient used for alcohol-free mouthwash. GMS Hyg. Infect. Control.

[115] do Nascimento C., Paulo D.F., Pita M.S., Pedrazzi V., de Albuquerque Junior R.F. (2015). Microbial diversity of the supra- and subgingival biofilm of healthy individuals after brushing with chlorhexidine- or silver-coated toothbrush bristles. Can. J. Microbiol..

[116] Mackevica A., Olsson M.E., Hansen S.F. (2017). The release of silver nanoparticles from commercial toothbrushes. J. Hazard. Mater..

[117] Gaillet S., Rouanet J.-M. (2015). Silver nanoparticles: Their potential toxic effects after oral exposure and underlying mechanisms—A review. Food Chem. Toxicol..

[118] Patil Y.M., Rajpathak S.N., Deobagkar D.D. (2019). Characterization and DNA methylation modulatory activity of gold nanoparticles synthesized by Pseudoalteromonas strain. J. Biosci..

[119] Liu R., Pei Q., Shou T., Zhang W., Hu J., Li W. (2019). Apoptotic effect of green synthesized gold nanoparticles from Curcuma wenyujin extract against human renal cell carcinoma A498 cells. Int. J. Nanomed..

[120] Chen M.-N., Chan C.-F., Huang S.-L., Lin Y.-S. (2019). Green biosynthesis of gold nanoparticles using Chenopodium formosanum shell extract and analysis of the particles’ antibacterial properties. J. Sci. Food Agric..

[121] Huang Q., Luo A., Jiang L., Zhou Y., Yang Y., Liu Q., Zhang C. (2019). Disinfection efficacy of green synthesized gold nanoparticles for medical disinfection applications. Afr. Health Sci..

[122] Verma A., Gautam S.P., Bansal K.K., Prabhakar N., Rosenholm J.M. (2019). Green Nanotechnology: Advancement in Phytoformulation Research. Medicines.

[123] Folorunso A., Akintelu S., Oyebamiji A.K., Ajayi S., Abiola B., Abdusalam I., Morakinyo A. (2019). Biosynthesis, characterization and antimicrobial activity of gold nanoparticles from leaf extracts of *Annona muricata*. J. Nanostruct. Chem..

[124] Keijok W.J., Pereira R.H.A., Alvarez L.A.C., Prado A.R., da Silva A.R., Ribeiro J., de Oliveira J.P., Guimarães M.C.C. (2019). Controlled biosynthesis of gold nanoparticles with *Coffea arabica* using factorial design. Sci. Rep..

[125] Raval C., Vyas K., Gandhi U., Patel B., Patel P. (2016). Nanotechnology in dentistry: A review. J. Adv. Med. Dent. Sci. Res..

[126] Katas H., Lim C.S., Nor Azlan A.Y.H., Buang F., Mh Busra M.F. (2019). Antibacterial activity of biosynthesized gold nanoparticles using biomolecules from *Lignosus rhinocerotis* and chitosan. Saudi Pharm. J..

[127] Yougbare S., Chang T.-K., Tan S.-H., Kuo J.-C., Hsu P.-H., Su C.-Y., Kuo T.-R. (2019). Antimicrobial Gold Nanoclusters: Recent Developments and Future Perspectives. Int. J. Mol. Sci..

[128] Makowski M., Silva Í.C., Pais do Amaral C., Gonçalves S., Santos N.C. (2019). Advances in Lipid and Metal Nanoparticles for Antimicrobial Peptide Delivery. Pharmaceutics.

[129] Naimi-Shamel N., Pourali P., Dolatabadi S. (2019). Green synthesis of gold nanoparticles using Fusarium oxysporum and antibacterial activity of its tetracycline conjugant. J. Mycol. Med.

[130] Fan Y., Pauer A.C., Gonzales A.A., Fenniri H. (2019). Enhanced antibiotic activity of ampicillin conjugated to gold nanoparticles on PEGylated rosette nanotubes. Int. J. Nanomed..

[131] Shamaila S., Zafar N., Riaz S., Sharif R., Nazir J., Naseem S. (2016). Gold Nanoparticles: An Efficient Antimicrobial Agent against Enteric Bacterial Human Pathogen. Nanomaterials.

[132] Connor E.E., Mwamuka J., Gole A., Murphy C.J., Wyatt M.D. (2005). Gold nanoparticles are taken up by human cells but do not cause acute cytotoxicity. Small Weinh. Bergstr. Ger..

[133] Elsome A.M., Hamilton-Miller J.M., Brumfitt W., Noble W.C. (1996). Antimicrobial activities in vitro and in vivo of transition element complexes containing gold(I) and osmium (VI). J. Antimicrob. Chemother..

[134] Novelli F., Recine M., Sparatore F., Juliano C. (1999). Gold(I) complexes as antimicrobial agents. Farm. Soc. Chim. Ital..

[135] Hernández-Sierra J.F., Ruiz F., Pena D.C.C., Martínez-Gutiérrez F., Martínez A.E., de Jesús Pozos Guillén A., Tapia-Pérez H., Castañón G.M. (2008). The antimicrobial sensitivity of Streptococcus mutans to nanoparticles of silver, zinc oxide, and gold. Nanomed. Nanotechnol. Biol. Med..

[136] Burdușel A.-C., Gherasim O., Grumezescu A.M., Mogoantă L., Ficai A., Andronescu E. (2018). Biomedical Applications of Silver Nanoparticles: An Up-to-Date Overview. Nanomaterials.

[137] AlKahtani R.N. (2018). The implications and applications of nanotechnology in dentistry: A review. Saudi Dent. J..

[138] Jia G., Zhi A., Lai P.F.H., Wang G., Xia Y., Xiong Z., Zhang H., Che N., Ai L. (2018). The oral microbiota—A mechanistic role for systemic diseases. Br. Dent. J..

[139] Gunduz N., Ceylan H., Guler M.O., Tekinay A.B. (2017). Intracellular Accumulation of Gold Nanoparticles Leads to Inhibition of Macropinocytosis to Reduce the Endoplasmic Reticulum Stress. Sci. Rep..

[140] Sung J.H., Ji J.H., Park J.D., Song M.Y., Song K.S., Ryu H.R., Yoon J.U., Jeon K.S., Jeong J., Han B.S. (2011). Subchronic inhalation toxicity of gold nanoparticles. Part. Fibre Toxicol..

[141] Rad S.S., Sani A.M., Mohseni S. (2019). Biosynthesis, characterization and antimicrobial activities of zinc oxide nanoparticles from leaf extract of *Mentha pulegium* (L.). Microb. Pathog..

[142] Ahmadi Shadmehri A., Namvar F., Miri H., Yaghmaei P., Nakhaei Moghaddam M. (2019). Assessment of antioxidant and antibacterial activities of Zinc Oxide nanoparticles, Graphene and Graphene decorated by Zinc Oxide nanoparticles. Int. J. Nano Dimens..

[143] Chemingui H., Missaoui T., Mzali J.C., Yildiz T., Konyar M., Smiri M., Saidi N., Hafiane A., Yatmaz H.C. (2019). Facile green synthesis of zinc oxide nanoparticles (ZnO NPs): Antibacterial and photocatalytic activities. Mater. Res. Express.

[144] Hajiashrafi S., Motakef Kazemi N. (2019). Preparation and evaluation of ZnO nanoparticles by thermal decomposition of MOF-5. Heliyon.

[145] Jones N., Ray B., Ranjit K.T., Manna A.C. (2008). Antibacterial activity of ZnO nanoparticle suspensions on a broad spectrum of microorganisms. FEMS Microbiol. Lett..

[146] Fang M., Chen J.-H., Xu X.-L., Yang P.-H., Hildebrand H.F. (2006). Antibacterial activities of inorganic agents on six bacteria associated with oral infections by two susceptibility tests. Int. J. Antimicrob. Agents.

[147] Ghaffari H., Tavakoli A., Moradi A., Tabarraei A., Bokharaei-Salim F., Zahmatkeshan M., Farahmand M., Javanmard D., Kiani S.J., Esghaei M. (2019). Inhibition of H1N1 influenza virus infection by zinc oxide nanoparticles: Another emerging application of nanomedicine. J. Biomed. Sci..

[148] Aldosari M.A., Darwish S.S., Adam M.A., Elmarzugi N.A., Ahmed S.M. (2019). Using ZnO nanoparticles in fungal inhibition and self-protection of exposed marble columns in historic sites. Archaeol. Anthropol. Sci..

[149] Jin S.-E., Jin H.-E. (2019). Synthesis, Characterization, and Three-Dimensional Structure Generation of Zinc Oxide-Based Nanomedicine for Biomedical Applications. Pharmaceutics.

[150] Mazitova G.T., Kienskaya K.I., Ivanova D.A., Belova I.A., Butorova I.A., Sardushkin M.V. (2019). Synthesis and Properties of Zinc Oxide Nanoparticles: Advances and Prospects. Rev. J. Chem..

[151] Kasraei S., Sami L., Hendi S., AliKhani M.-Y., Rezaei-Soufi L., Khamverdi Z. (2014). Antibacterial properties of composite resins incorporating silver and zinc oxide nanoparticles on Streptococcus mutans and Lactobacillus. Restor. Dent. Endod..

[152] Sirelkhatim A., Mahmud S., Seeni A., Kaus N.H.M., Ann L.C., Bakhori S.K.M., Hasan H., Mohamad D. (2015). Review on Zinc Oxide Nanoparticles: Antibacterial Activity and Toxicity Mechanism. Nano-Micro Lett..

[153] Brayner R., Ferrari-Iliou R., Brivois N., Djediat S., Benedetti M.F., Fiévet F. (2006). Toxicological impact studies based on Escherichia coli bacteria in ultrafine ZnO nanoparticles colloidal medium. Nano Lett..

[154] Pranjali P., Meher M.K., Raj R., Prasad N., Poluri K.M., Kumar D., Guleria A. (2019). Physicochemical and Antibacterial Properties of PEGylated Zinc Oxide Nanoparticles Dispersed in Peritoneal Dialysis Fluid. ACS Omega.

[155] Hirota K., Sugimoto M., Kato M., Tsukagoshi K., Tanigawa T., Sugimoto H. (2010). Preparation of zinc oxide ceramics with a sustainable antibacterial activity under dark conditions. Ceram. Int..

[156] Memarzadeh K., Vargas M., Huang J., Fan J., Allaker R. (2011). Nano Metallic-Oxides as Antimicrobials for Implant Coatings. Key Eng. Mater..

[157] Vargas-Reus M.A., Memarzadeh K., Huang J., Ren G.G., Allaker R.P. (2012). Antimicrobial activity of nanoparticulate metal oxides against peri-implantitis pathogens. Int. J. Antimicrob. Agents.

[158] Osorio R., Yamauti M., Osorio E., Ruiz-Requena M., Pashley D., Tay F., Toledano M. (2011). Zinc reduces collagen degradation in demineralized human dentin explants. J. Dent..

[159] Nagajyothi P.C., Cha S.J., Yang I.J., Sreekanth T.V.M., Kim K.J., Shin H.M. (2015). Antioxidant and anti-inflammatory activities of zinc oxide nanoparticles synthesized using Polygala tenuifolia root extract. J. Photochem. Photobiol. B.

[160] Mahmood A., Mneimne M., Zou L.F., Hill R.G., Gillam D.G. (2014). Abrasive wear of enamel by bioactive glass-based toothpastes. Am. J. Dent..

[161] Takatsuka T., Tanaka K., Iijima Y. (2005). Inhibition of dentine demineralization by zinc oxide: In vitro and in situ studies. Dent. Mater..

[162] Lynch E., Brauer D.S., Karpukhina N., Gillam D.G., Hill R.G. (2012). Multi-component bioactive glasses of varying fluoride content for treating dentin hypersensitivity. Dent. Mater..

[163] Burguera-Pascu M., Rodríguez-Archilla A., Baca P. (2007). Substantivity of zinc salts used as rinsing solutions and their effect on the inhibition of Streptococcus mutans. J. Trace Elem. Med. Biol. Organ Soc. Miner. Trace Elem. GMS.

[164] Almoudi M.M., Hussein A.S., Abu Hassan M.I., Mohamad Zain N. (2018). A systematic review on antibacterial activity of zinc against Streptococcus mutans. Saudi Dent. J..

[165] Ahrari F., Eslami N., Rajabi O., Ghazvini K., Barati S. (2015). The antimicrobial sensitivity of *Streptococcus mutans* and *Streptococcus sangius* to colloidal solutions of different nanoparticles applied as mouthwashes. Dent. Res. J..

[166] Ghosh S., Goudar V.S., Padmalekha K.G., Bhat S.V., Indi S.S., Vasan H.N. (2012). ZnO/Ag nanohybrid: Synthesis, characterization, synergistic antibacterial activity and its mechanism. RSC Adv..

[167] Kachoei M.Y., Divband B., Tabriz F.D., Helali Z.N., Esmailzadeh M. (2018). A comparative study of antibacterial effects of mouthwashes containing Ag/ZnO or ZnO nanoparticles with chlorhexidine and investigation of their cytotoxicity. Nanomed. J..

[168] Eslami N., Ahrari F., Rajabi O., Zamani R. (2015). The staining effect of different mouthwashes containing nanoparticles on dental enamel. J. Clin. Exp. Dent..

[169] Liu W., Su P., Chen S., Wang N., Ma Y., Liu Y., Wang J., Zhang Z., Li H., Webster T.J. (2014). Synthesis of TiO_2_ nanotubes with ZnO nanoparticles to achieve antibacterial properties and stem cell compatibility. Nanoscale.

[170] Kulkarni V., Palled V., Hiregoudar S., Prakash K., Maski D., Lendra S. (2019). Bio-Synthesis and Characterization of Titanium Dioxide Nanoparticles (TiO_2_) Using Azadirachta indica Leaf (Neem Leaf) Extract. Int. J. Curr. Microbiol. Appl. Sci..

[171] Madadi Z., Soltanieh M., Bagheri Lotfabad T., Nazari S.N. (2019). Green synthesis of titanium dioxide nanoparticles with Glycyrrhiza glabra and their photocatalytic activity. Asian J. Green Chem..

[172] Kaur H., Kaur S., Singh J., Rawat M., Kumar S. (2019). Expanding horizon: Green synthesis of TiO_2_ nanoparticles using Carica papaya leaves for photocatalysis application. Mater. Res. Express.

[173] Swathi N., Sandhiya D., Rajeshkumar S., Lakshmi T. (2019). Green synthesis of titanium dioxide nanoparticles using Cassia fistula and its antibacterial activity. Int. J. Res. Pharm. Sci..

[174] de Dicastillo C.L., Patiño C., Galotto M.J., Vásquez-Martínez Y., Torrent C., Alburquenque D., Pereira A., Escrig J. (2019). Novel hollow titanium dioxide nanospheres with antimicrobial activity against resistant bacteria. Beilstein J. Nanotechnol..

[175] Azizi-Lalabadi M., Ehsani A., Divband B., Alizadeh-Sani M. (2019). Antimicrobial activity of Titanium dioxide and Zinc oxide nanoparticles supported in 4A zeolite and evaluation the morphological characteristic. Sci. Rep..

[176] Akhtar S., Shahzad K., Mushtaq S., Ali I., Rafe M.H., Fazal-ul-Karim S.M. (2019). Antibacterial and antiviral potential of colloidal Titanium dioxide (TiO_2_) nanoparticles suitable for biological applications. Mater. Res. Express.

[177] Zhao Z., Zhang X., Zhang G., Liu Z., Qu D., Miao X., Feng P., Sun Z. (2015). Effect of defects on photocatalytic activity of rutile TiO_2_ nanorods. Nano Res..

[178] Feng X., Pan F., Zhao H., Deng W., Zhang P., Zhou H.-C., Li Y. (2018). Atomic layer deposition enabled MgO surface coating on porous TiO_2_ for improved CO_2_ photoreduction. Appl. Catal. B Environ..

[179] Esfandiari N., Simchi A., Bagheri R. (2014). Size tuning of Ag-decorated TiO_2_ nanotube arrays for improved bactericidal capacity of orthopedic implants. J. Biomed. Mater. Res. A.

[180] Cushing B.L., Kolesnichenko V.L., O’Connor C.J. (2004). Recent Advances in the Liquid-Phase Syntheses of Inorganic Nanoparticles. Chem. Rev..

[181] Hassan H., Omoniyi K.I., Okibe F.G., Nuhu A.A., Echioba E.G. (2019). Evaluation of Antibacterial Potential of Biosynthesized Plant Leave Extract Mediated Titanium Oxide Nanoparticles using Hypheae Thiebeace and Anannos Seneglensis. J. Appl. Sci. Environ. Manag..

[182] Mohammadi S., Mohammadi P., Hosseinkhani S., Shipour R. (2013). Antifungal Activity of TiO2 nanoparticles and EDTA on Candida albicans Biofilms. Infect. Epidemiol. Med..

[183] Maness P.-C., Smolinski S., Blake D.M., Huang Z., Wolfrum E.J., Jacoby W.A. (1999). Bactericidal Activity of Photocatalytic TiO_2_ Reaction: Toward an Understanding of Its Killing Mechanism. Appl. Environ. Microbiol..

[184] Tsuang Y.-H., Sun J.-S., Huang Y.-C., Lu C.-H., Chang W.H.-S., Wang C.-C. (2008). Studies of photokilling of bacteria using titanium dioxide nanoparticles. Artif. Organs.

[185] Wysocka I., Kowalska E., Ryl J., Nowaczyk G., Zielińska-Jurek A. (2019). Morphology, Photocatalytic and Antimicrobial Properties of TiO_2_ Modified with Mono- and Bimetallic Copper, Platinum and Silver Nanoparticles. Nanomaterials.

[186] Chambers C., Stewart S.B., Su B., Jenkinson H.F., Sandy J.R., Ireland A.J. (2017). Silver doped titanium dioxide nanoparticles as antimicrobial additives to dental polymers. Dent. Mater..

[187] Durango-Giraldo G., Cardona A., Zapata J.F., Santa J.F., Buitrago-Sierra R. (2019). Titanium dioxide modified with silver by two methods for bactericidal applications. Heliyon.

[188] Komatsu O., Nishida H., Sekino T., Yamamoto K. (2014). Application of Titanium Dioxide Nanotubes to Tooth Whitening. Nano Biomed..

[189] Iavicoli I., Leso V., Fontana L., Bergamaschi A. (2011). Toxicological effects of titanium dioxide nanoparticles: A review of in vitro mammalian studies. Eur. Rev. Med. Pharmacol. Sci..

[190] Iavicoli I., Leso V., Bergamaschi A. Toxicological Effects of Titanium Dioxide Nanoparticles: A Review of In Vivo Studies. https://www.hindawi.com/journals/jnm/2012/964381/.

[191] Shi H., Magaye R., Castranova V., Zhao J. (2013). Titanium dioxide nanoparticles: A review of current toxicological data. Part. Fibre Toxicol..

[192] Geraets L., Oomen A.G., Krystek P., Jacobsen N.R., Wallin H., Laurentie M., Verharen H.W., Brandon E.F.A., de Jong W.H. (2014). Tissue distribution and elimination after oral and intravenous administration of different titanium dioxide nanoparticles in rats. Part. Fibre Toxicol..

[193] Rompelberg C., Heringa M.B., van Donkersgoed G., Drijvers J., Roos A., Westenbrink S., Peters R., van Bemmel G., Brand W., Oomen A.G. (2016). Oral intake of added titanium dioxide and its nanofraction from food products, food supplements and toothpaste by the Dutch population. Nanotoxicology.

[194] Baranowska-Wójcik E., Szwajgier D., Oleszczuk P., Winiarska-Mieczan A. (2019). Effects of Titanium Dioxide Nanoparticles Exposure on Human Health—A Review. Biol. Trace Elem. Res..

[195] Buazar F., Sweidi S., Badri M., Kroushawi F. (2019). Biofabrication of highly pure copper oxide nanoparticles using wheat seed extract and their catalytic activity: A mechanistic approach. Green Process. Synth..

[196] Khatami M., Varma R.S., Heydari M., Peydayesh M., Sedighi A., Askari H.A., Rohani M., Baniasadi M., Arkia S., Seyedi F. (2019). Copper Oxide Nanoparticles Greener Synthesis Using Tea and its Antifungal Efficiency on Fusarium solani. Geomicrobiol. J..

[197] Tavakoli S., Kharaziha M., Ahmadi S. (2019). Green synthesis and morphology dependent antibacterial activity of copper oxide nanoparticles. J. Nanostruct..

[198] Mohamed Hamouda I. (2012). Current perspectives of nanoparticles in medical and dental biomaterials. J. Biomed. Res..

[199] Ren G., Hu D., Cheng E.W.C., Vargas-Reus M.A., Reip P., Allaker R.P. (2009). Characterisation of copper oxide nanoparticles for antimicrobial applications. Int. J. Antimicrob. Agents.

[200] Amiri M., Etemadifar Z., Daneshkazemi A., Nateghi M. (2017). Antimicrobial Effect of Copper Oxide Nanoparticles on Some Oral Bacteria and Candida Species. J. Dent. Biomater..

[201] Varoni E., Tarce M., Lodi G., Carrassi A. (2012). Chlorhexidine (CHX) in dentistry: State of the art. Minerva Stomatol..

[202] Barbour M.E., Maddocks S.E., Wood N.J., Collins A.M. (2013). Synthesis, characterization, and efficacy of antimicrobial chlorhexidine hexametaphosphate nanoparticles for applications in biomedical materials and consumer products. Int. J. Nanomed..

[203] Zhu B., Macleod L.C., Kitten T., Xu P. (2018). Streptococcus sanguinis biofilm formation & interaction with oral pathogens. Future Microbiol..

[204] Seneviratne C.J., Leung K.C.-F., Wong C.-H., Lee S.-F., Li X., Leung P.C., Lau C.B.S., Wat E., Jin L. (2014). Nanoparticle-Encapsulated Chlorhexidine against Oral Bacterial Biofilms. PLoS ONE.

[205] Liu Y., Naha P.C., Hwang G., Kim D., Huang Y., Simon-Soro A., Jung H.-I., Ren Z., Li Y., Gubara S. (2018). Topical ferumoxytol nanoparticles disrupt biofilms and prevent tooth decay in vivo via intrinsic catalytic activity. Nat. Commun..

[206] Ikemoto S., Sugimura K., Yoshida N., Yasumoto R., Wada S., Yamamoto K., Kishimoto T. (2000). Antitumor effects of Scutellariae radix and its components baicalein, baicalin, and wogonin on bladder cancer cell lines. Urology.

[207] Eid Abdelmagyd H.A., Ram Shetty D.S., Musa Musleh Al-Ahmari D.M. (2019). Herbal medicine as adjunct in periodontal therapies—A review of clinical trials in past decade. J. Oral Biol. Craniofacial Res..

[208] Luo W., Wang C.-Y., Jin L. (2012). Baicalin downregulates Porphyromonas gingivalis lipopolysaccharide-upregulated IL-6 and IL-8 expression in human oral keratinocytes by negative regulation of TLR signaling. PLoS ONE.

[209] Sheng W.S., Hsueh P.R., Hung C.C., Teng L.J., Chen Y.C., Luh K.T. (2001). Clinical features of patients with invasive Eikenella corrodens infections and microbiological characteristics of the causative isolates. Eur. J. Clin. Microbiol. Infect. Dis..

[210] Qing L.-S., Xiong J., Xue Y., Liu Y.-M., Guang B., Ding L.-S., Liao X. (2011). Using baicalin-functionalized magnetic nanoparticles for selectively extracting flavonoids from Rosa chinensis. J. Sep. Sci..

[211] Wang L., Zhang H., Chen B., Xia G., Wang S., Cheng J., Shao Z., Gao C., Bao W., Tian L. (2012). Effect of magnetic nanoparticles on apoptosis and cell cycle induced by wogonin in Raji cells. Int. J. Nanomed..

[212] Nipun Babu V., Kannan S. (2012). Enhanced delivery of baicalein using cinnamaldehyde cross-linked chitosan nanoparticle inducing apoptosis. Int. J. Biol. Macromol..

[213] Leung K.C.-F., Seneviratne C.J., Li X., Leung P.C., Lau C.B.S., Wong C.-H., Pang K.Y., Wong C.W., Wat E., Jin L. (2016). Synergistic Antibacterial Effects of Nanoparticles Encapsulated with Scutellaria baicalensis and Pure Chlorhexidine on Oral Bacterial Biofilms. Nanomaterials.

[214] Paul W., Sharma C.P. (2000). Chitosan, a drug carrier for the 21st century: A review. T P Pharma Sci..

[215] Dash M., Chiellini F., Ottenbrite R.M., Chiellini E. (2011). Chitosan—A versatile semi-synthetic polymer in biomedical applications. Prog. Polym. Sci..

[216] Costa T.H.R., de Figueiredo Neto J.A., de Oliveira A.E.F., e Maia M.D.F.L., de Almeida A.L. (2014). Association between chronic apical periodontitis and coronary artery disease. J. Endod..

[217] Costa E.M., Silva S., Costa M.R., Pereira M., Campos D.A., Odila J., Madureira A.R., Cardelle-Cobas A., Tavaria F.K., Rodrigues A.S. (2014). Chitosan mouthwash: Toxicity and in vivo validation. Carbohydr. Polym..

[218] Sámano-Valencia C., Martínez-Castañón G.A., Martínez-Martínez R.E., Loyola-Rodríguez J.P., Reyes-Macías J.F., Ortega-Zarzosa G., Niño-Martínez N. (2013). Bactericide efficiency of a combination of chitosan gel with silver nanoparticles. Mater. Lett..

[219] Mohire N.C., Yadav A.V. (2010). Chitosan-based polyherbal toothpaste: As novel oral hygiene product. Indian J. Dent. Res..

[220] Schlueter N., Klimek J., Ganss C. (2013). Randomised in situ study on the efficacy of a tin/chitosan toothpaste on erosive-abrasive enamel loss. Caries Res..

[221] Schlueter N., Klimek J., Ganss C. (2014). Effect of a chitosan additive to a Sn2+-containing toothpaste on its anti-erosive/anti-abrasive efficacy—A controlled randomised in situ trial. Clin. Oral Investig..

[222] Uysal T., Akkurt M.D., Amasyali M., Ozcan S., Yagci A., Basak F., Sagdic D. (2011). Does a chitosan-containing dentifrice prevent demineralization around orthodontic brackets?. Angle Orthod..

[223] Featherstone J.D. (2000). The science and practice of caries prevention. J. Am. Dent. Assoc..

[224] Featherstone J.D.B. (2004). The continuum of dental caries—Evidence for a dynamic disease process. J. Dent. Res..

[225] Deng D.M., ten Cate J.M. (2004). Demineralization of dentin by Streptococcus mutans biofilms grown in the constant depth film fermentor. Caries Res..

[226] Totiam P., González-Cabezas C., Fontana M.R., Zero D.T. (2007). A new in vitro model to study the relationship of gap size and secondary caries. Caries Res..

[227] Horst J.A., Tanzer J.M., Milgrom P.M. (2018). Fluorides and Other Preventive Strategies for Tooth Decay. Dent. Clin. N. Am..

[228] Mahoney E.K., Kilpatrick N.M. (2003). Dental erosion: Part 1. Aetiology and prevalence of dental erosion. N. Z. Dent. J..

[229] Davari A., Ataei E., Assarzadeh H. (2013). Dentin Hypersensitivity: Etiology, Diagnosis and Treatment; A Literature Review. J. Dent..

[230] Dai Z., Liu M., Ma Y., Cao L., Xu H.H.K., Zhang K., Bai Y. Effects of Fluoride and Calcium Phosphate Materials on Remineralization of Mild and Severe White Spot Lesions. https://www.hindawi.com/journals/bmri/2019/1271523/.

[231] Pepla E., Besharat L.K., Palaia G., Tenore G., Migliau G. (2014). Nano-hydroxyapatite and its applications in preventive, restorative and regenerative dentistry: A review of literature. Ann. Stomatol. (Roma).

[232] Nozari A., Ajami S., Rafiei A., Niazi E. (2017). Impact of Nano Hydroxyapatite, Nano Silver Fluoride and Sodium Fluoride Varnish on Primary Teeth Enamel Remineralization: An In Vitro Study. J. Clin. Diagn. Res..

[233] Vandiver J., Dean D., Patel N., Bonfield W., Ortiz C. (2005). Nanoscale variation in surface charge of synthetic hydroxyapatite detected by chemically and spatially specific high-resolution force spectroscopy. Biomaterials.

[234] Li L., Pan H., Tao J., Xu X., Mao C., Gu X., Tang R. (2008). Repair of enamel by using hydroxyapatite nanoparticles as the building blocks. J. Mater. Chem..

[235] Swarup J.S., Rao A. (2012). Enamel surface remineralization: Using synthetic nanohydroxyapatite. Contemp. Clin. Dent..

[236] Roveri N., Battistella E., Bianchi C.L., Foltran I., Foresti E., Iafisco M., Lelli M., Naldoni A., Palazzo B., Rimondini L. Surface Enamel Remineralization: Biomimetic Apatite Nanocrystals and Fluoride Ions Different Effects. https://www.hindawi.com/journals/jnm/2009/746383/.

[237] Chen H., Clarkson B.H., Sun K., Mansfield J.F. (2005). Self-assembly of synthetic hydroxyapatite nanorods into an enamel prism-like structure. J. Colloid Interface Sci..

[238] Vano M., Derchi G., Barone A., Covani U. (2014). Effectiveness of nano-hydroxyapatite toothpaste in reducing dentin hypersensitivity: A double-blind randomized controlled trial. Quintessence Int. Berl. Ger..

[239] Vano M., Derchi G., Barone A., Pinna R., Usai P., Covani U. (2018). Reducing dentine hypersensitivity with nano-hydroxyapatite toothpaste: A double-blind randomized controlled trial. Clin. Oral Investig..

[240] Jena A., Kala S., Shashirekha G. (2017). Comparing the effectiveness of four desensitizing toothpastes on dentinal tubule occlusion: A scanning electron microscope analysis. J. Conserv. Dent..

[241] Ramis J.M., Coelho C.C., Córdoba A., Quadros P.A., Monjo M. (2018). Safety Assessment of Nano-Hydroxyapatite as an Oral Care Ingredient according to the EU Cosmetics Regulation. Cosmetics.

[242] Kani T., Kani M., Isozaki A., Shintani H., Ohashi T., Tokumoto T. (1989). Effect to Apatite-containing Dentifrices on Dental Caries in School Children. J. Dent. Health.

[243] Ebadifar A., Nomani M., Fatemi S.A. (2017). Effect of nano-hydroxyapatite toothpaste on microhardness ofartificial carious lesions created on extracted teeth. J. Dent. Res. Dent. Clin. Dent. Prospects.

[244] Hiller K.-A., Buchalla W., Grillmeier I., Neubauer C., Schmalz G. (2018). In vitro effects of hydroxyapatite containing toothpastes on dentin permeability after multiple applications and ageing. Sci. Rep..

[245] Mielczarek A., Michalik J. (2014). The effect of nano-hydroxyapatite toothpaste on enamel surface remineralization. An in vitro study. Am. J. Dent..

[246] Madhusudanan P., SV P., Pillai R., Varghese N., George S., Antony A. (2018). Comparative Evaluation of Surface Microhardness of Artificially Demineralized Human Enamel with Nano hydroxyapatite, Calcium Phosphate, and Potassium Nitrate Remineralizing Agents: An In Vitro Study. Conserv. Dent. Endod. J..

[247] Kulal R., Jayanti I., Sambashivaiah S., Bilchodmath S. (2016). An In-vitro Comparison of Nano Hydroxyapatite, Novamin and Proargin Desensitizing Toothpastes—A SEM Study. J. Clin. Diagn. Res..

[248] Orsini G., Procaccini M., Manzoli L., Giuliodori F., Lorenzini A., Putignano A. (2010). A double-blind randomized-controlled trial comparing the desensitizing efficacy of a new dentifrice containing carbonate/hydroxyapatite nanocrystals and a sodium fluoride/potassium nitrate dentifrice. J. Clin. Periodontol..

[249] Orsini G., Procaccini M., Manzoli L., Sparabombe S., Tiriduzzi P., Bambini F., Putignano A. (2013). A 3-day randomized clinical trial to investigate the desensitizing properties of three dentifrices. J. Periodontol..

[250] Gopinath N.M., John J., Nagappan N., Prabhu S., Kumar E.S. (2015). Evaluation of Dentifrice Containing Nano-hydroxyapatite for Dentinal Hypersensitivity: A Randomized Controlled Trial. J. Int. Oral Health.

[251] Jena A., Shashirekha G. (2015). Comparison of efficacy of three different desensitizing agents for in-office relief of dentin hypersensitivity: A 4 weeks clinical study. J. Conserv. Dent..

[252] Bossù M., Saccucci M., Salucci A., Di Giorgio G., Bruni E., Uccelletti D., Sarto M.S., Familiari G., Relucenti M., Polimeni A. (2019). Enamel remineralization and repair results of Biomimetic Hydroxyapatite toothpaste on deciduous teeth: An effective option to fluoride toothpaste. J. Nanobiotechnol..

[253] Browning W.D., Cho S.D., Deschepper E.J. (2012). Effect of a nano-hydroxyapatite paste on bleaching-related tooth sensitivity. J. Esthet. Restor. Dent..

[254] Jin J., Xu X., Lai G., Kunzelmann K.-H. (2013). Efficacy of tooth whitening with different calcium phosphate-based formulations. Eur. J. Oral Sci..

[255] Niwa M., Sato T., Li W., Aoki H., Aoki H., Daisaku T. (2001). Polishing and whitening properties of toothpaste containing hydroxyapatite. J. Mater. Sci. Mater. Med..

[256] Marinho V.C., Higgins J.P., Sheiham A., Logan S. (2003). Fluoride toothpastes for preventing dental caries in children and adolescents. Cochrane Database Syst. Rev..

[257] Evans R.W., Dennison P.J. (2009). The Caries Management System: An evidence-based preventive strategy for dental practitioners. Application for children and adolescents. Aust. Dent. J..

[258] Pendrys D.G., Stamm J.W. (1990). Relationship of total fluoride intake to beneficial effects and enamel fluorosis. J. Dent. Res..

[259] Tschoppe P., Zandim D.L., Martus P., Kielbassa A.M. (2011). Enamel and dentine remineralization by nano-hydroxyapatite toothpastes. J. Dent..

[260] Manchery N., John J., Nagappan N., Subbiah G.K., Premnath P. (2019). Remineralization potential of dentifrice containing nanohydroxyapatite on artificial carious lesions of enamel: A comparative in vitro study. Dent. Res. J..

[261] Colombo M., Beltrami R., Rattalino D., Mirando M., Chiesa M., Poggio C. (2016). Protective effects of a zinc-hydroxyapatite toothpaste on enamel erosion: SEM study. Ann. Stomatol. (Roma).

[262] Pajor K., Pajchel L., Kolmas J. (2019). Hydroxyapatite and Fluorapatite in Conservative Dentistry and Oral Implantology—A Review. Materials.

[263] Hannig C., Basche S., Burghardt T., Al-Ahmad A., Hannig M. (2013). Influence of a mouthwash containing hydroxyapatite microclusters on bacterial adherence in situ. Clin. Oral Investig..

[264] Hegazy S.A., Salama R.I. (2016). Antiplaque and remineralizing effects of Biorepair mouthwash: A comparative clinical trial. Pediatr. Dent. J..

[265] Kensche A., Holder C., Basche S., Tahan N., Hannig C., Hannig M. (2017). Efficacy of a mouthrinse based on hydroxyapatite to reduce initial bacterial colonisation in situ. Arch. Oral Biol..

[266] Palmieri C., Magi G., Orsini G., Putignano A., Facinelli B. (2013). Antibiofilm activity of zinc-carbonate hydroxyapatite nanocrystals against Streptococcus mutans and mitis group streptococci. Curr. Microbiol..

[267] Lata S., Varghese N.O., Varughese J.M. (2010). Remineralization potential of fluoride and amorphous calcium phosphate-casein phospho peptide on enamel lesions: An in vitro comparative evaluation. J. Conserv. Dent..

[268] Ceci M., Mirando M., Beltrami R., Chiesa M., Poggio C. (2015). Protective effect of casein phosphopeptide-amorphous calcium phosphate on enamel erosion: Atomic force microscopy studies. Scanning.

[269] Hegde M.N., Moany A. (2012). Remineralization of enamel subsurface lesions with casein phosphopeptide-amorphous calcium phosphate: A quantitative energy dispersive X-ray analysis using scanning electron microscopy: An in vitro study. J. Conserv. Dent..

[270] White A.J., Gracia L.H., Barbour M.E. (2011). Inhibition of dental erosion by casein and casein-derived proteins. Caries Res..

[271] Rao S.K., Bhat G.S., Aradhya S., Devi A., Bhat M. (2009). Study of the efficacy of toothpaste containing casein phosphopeptide in the prevention of dental caries: A randomized controlled trial in 12- to 15-year-old high caries risk children in Bangalore, India. Caries Res..

[272] Poggio C., Lombardini M., Colombo M., Bianchi S. (2010). Impact of two toothpastes on repairing enamel erosion produced by a soft drink: An AFM in vitro study. J. Dent..

[273] Poggio C., Lombardini M., Vigorelli P., Ceci M. (2013). Analysis of dentin/enamel remineralization by a CPP-ACP paste: AFM and SEM study. Scanning.

[274] Grewal N., Kudupudi V., Grewal S. (2013). Surface remineralization potential of casein phosphopeptide-amorphous calcium phosphate on enamel eroded by cola-drinks: An in-situ model study. Contemp. Clin. Dent..

[275] Cunha A.G.G., De Vasconcelos A.A.M., Borges B.C.D., Vitoriano J.D.O., Alves-Junior C., Machado C.T., Dos Santos A.J.S. (2012). Efficacy of in-office bleaching techniques combined with the application of a casein phosphopeptide-amorphous calcium phosphate paste at different moments and its influence on enamel surface properties. Microsc. Res. Tech..

[276] Sharma A., Rao A., Shenoy R., Suprabha B.S. (2017). Comparative evaluation of Nano-hydroxyapatite and casein Phosphopeptide-amorphous calcium phosphate on the remineralization potential of early enamel lesions: An in vitro study. J. Orofac. Sci..

[277] Reynolds E.C. (1998). Anticariogenic complexes of amorphous calcium phosphate stabilized by casein phosphopeptides: A review. Spec. Care Dent..

[278] Oliveira G.M.S., Ritter A.V., Heymann H.O., Swift E., Donovan T., Brock G., Wright T. (2014). Remineralization effect of CPP-ACP and fluoride for white spot lesions in vitro. J. Dent..

[279] Shetty S., Hegde M.N., Bopanna T.P. (2014). Enamel remineralization assessment after treatment with three different remineralizing agents using surface microhardness: An in vitro study. J. Conserv. Dent..

[280] Elgamily H., Safwat E., Soliman Z., Salama H., El-Sayed H., Anwar M. (2019). Antibacterial and Remineralization Efficacy of Casein Phosphopeptide, Glycomacropeptide Nanocomplex, and Probiotics in Experimental Toothpastes: An In Vitro Comparative Study. Eur. J. Dent..

[281] Dai L.L., Mei M.L., Chu C.H., Lo E.C.M. (2019). Mechanisms of Bioactive Glass on Caries Management: A Review. Materials.

[282] Jones J.R. (2013). Review of bioactive glass: From Hench to hybrids. Acta Biomater..

[283] Ramashetty Prabhakar A., Arali V. (2009). Comparison of the Remineralizing Effects of Sodium Fluoride and Bioactive Glass Using Bioerodible Gel Systems. J. Dent. Res. Dent. Clin. Dent. Prospects.

[284] Vollenweider M., Brunner T.J., Knecht S., Grass R.N., Zehnder M., Imfeld T., Stark W.J. (2007). Remineralization of human dentin using ultrafine bioactive glass particles. Acta Biomater..

[285] Zhang Y., Wang Z., Jiang T., Wang Y. (2019). Biomimetic regulation of dentine remineralization by amino acid in vitro. Dent. Mater..

[286] Burwell A., Jennings D., Muscle D., Greenspan D.C. (2010). NovaMin and dentin hypersensitivity--in vitro evidence of efficacy. J. Clin. Dent..

[287] Shivaprasad B.M., Padmavati P., Nehal N.S. (2014). Chair Side Application of NovaMin for the Treatment of Dentinal Hypersensitivity- A Novel Technique. J. Clin. Diagn. Res..

[288] Fiume E., Barberi J., Verné E., Baino F. (2018). Bioactive Glasses: From Parent 45S5 Composition to Scaffold-Assisted Tissue-Healing Therapies. J. Funct. Biomater..

[289] Skallevold H.E., Rokaya D., Khurshid Z., Zafar M.S. (2019). Bioactive Glass Applications in Dentistry. Int. J. Mol. Sci..

[290] Sheng X.-Y., Gong W.-Y., Hu Q., Chen X., Dong Y.-M. (2016). Mineral formation on dentin induced by nano-bioactive glass. Chin. Chem. Lett..

[291] Aras A., Celenk S., Dogan M.S., Bardakci E. (2019). Comparative evaluation of combined remineralization agents on demineralized tooth surface. Niger. J. Clin. Pract..

[292] Rajendran R., Kunjusankaran R.N., Sandhya R., Anilkumar A., Santhosh R., Patil S.R., Rajendran R., Kunjusankaran R.N., Sandhya R., Anilkumar A. (2019). Comparative Evaluation of Remineralizing Potential of a Paste Containing Bioactive Glass and a Topical Cream Containing Casein Phosphopeptide-Amorphous Calcium Phosphate: An in Vitro Study. Pesqui. Bras. Em Odontopediatria E Clínica Integr..

[293] Sauro S., Thompson I., Watson T.F. (2011). Effects of common dental materials used in preventive or operative dentistry on dentin permeability and remineralization. Oper. Dent..

[294] Farooq I., Majeed A., Alshwaimi E., Almas K. (2019). Title: Efficacy of a novel fluoride containing bioactive glass based dentifrice in remineralizing artificially induced demineralization in human enamel. Fluoride.

[295] Patel V.R., Shettar L., Thakur S., Gillam D., Kamala D.N. (2019). A randomised clinical trial on the efficacy of 5% fluorocalcium phosphosilicate-containing novel bioactive glass toothpaste. J. Oral Rehabil..

[296] Xu Y.-T., Wu Q., Chen Y.-M., Smales R.J., Shi S.-Y., Wang M.-T. (2015). Antimicrobial effects of a bioactive glass combined with fluoride or triclosan on Streptococcus mutans biofilm. Arch. Oral Biol..

[297] Rajan R., Krishnan R., Bhaskaran B., Kumar S.V. (2015). A Polarized Light Microscopic Study to Comparatively evaluate Four Remineralizing Agents on Enamel viz CPP-ACPF, ReminPro, SHY-NM and Colgate Strong Teeth. Int. J. Clin. Pediatr. Dent..

[298] Soares R., De Ataide I.D.N., Fernandes M., Lambor R. (2017). Assessment of Enamel Remineralisation after Treatment with Four Different Remineralising Agents: A Scanning Electron Microscopy (SEM) Study. J. Clin. Diagn. Res..

[299] Ali S., Farooq I., Iqbal K. (2014). A review of the effect of various ions on the properties and the clinical applications of novel bioactive glasses in medicine and dentistry. Saudi Dent. J..

[300] Saffarpour M., Mohammadi M., Tahriri M., Zakerzadeh A. (2017). Efficacy of Modified Bioactive Glass for Dentin Remineralization and Obstruction of Dentinal Tubules. J. Dent. Tehran Iran.

[301] Borm P.J.A., Robbins D., Haubold S., Kuhlbusch T., Fissan H., Donaldson K., Schins R., Stone V., Kreyling W., Lademann J. (2006). The potential risks of nanomaterials: A review carried out for ECETOC. Part. Fibre Toxicol..

[302] Adverse Effects of Engineered Nanomaterials—2nd Edition. https://www.elsevier.com/books/adverse-effects-of-engineered-nanomaterials/fadeel/978-0-12-809199-9.

[303] SCCS (Scientific Committee on Consumer Safety) (2019). Guidance on the Safety Assessment of Nanomaterials in Cosmetics.

[304] Halla N., Fernandes I.P., Heleno S.A., Costa P., Boucherit-Otmani Z., Boucherit K., Rodrigues A.E., Ferreira I.C.F.R., Barreiro M.F. (2018). Cosmetics Preservation: A Review on Present Strategies. Molecules.

[305] SCCS (Scientific Committee on Consumer Safety) (2012). Notes of Guidance for the Testing of Cosmetic Substances and Their Safety Evaluation.

[306] Oberdörster G. (2000). Determinants of the pathogenicity of man-made vitreous fibers (MMVF). Int. Arch. Occup. Environ. Health.

[307] Utembe W., Potgieter K., Stefaniak A.B., Gulumian M. (2015). Dissolution and biodurability: Important parameters needed for risk assessment of nanomaterials. Part. Fibre Toxicol..

[308] Hall B., Steiling W., Safford B., Coroama M., Tozer S., Firmani C., McNamara C., Gibney M. (2011). European consumer exposure to cosmetic products, a framework for conducting population exposure assessments Part 2. Food Chem. Toxicol..

[309] Gomez-Berrada M.-P., Ficheux A.-S., Boudières I., Chiter M., Rielland A., De Javel D., Roudot A.-C., Ferret P.-J. (2018). Consumption and exposure assessment to toothpaste in French families. Food Chem. Toxicol..

[310] Bernard A., Dornic N., Roudot A., Ficheux A. (2018). Probabilistic exposure assessment to face and oral care cosmetic products by the French population. Food Chem. Toxicol..

[311] Strittholt C.A., McMillan D.A., He T., Baker R.A., Barker M.L. (2016). A randomized clinical study to assess ingestion of dentifrice by children. Regul. Toxicol. Pharmacol..

